# ScientISST CORE: A novel hardware development platform for biomedical engineering

**DOI:** 10.1016/j.ohx.2025.e00630

**Published:** 2025-02-17

**Authors:** Leonor Pereira, Francisco de Melo, Frederico Almeida Santos, Afonso Fortes Ferreira, Hugo Plácido da Silva

**Affiliations:** a Instituto Superior Técnico, Universidade de Lisboa, 1050-049 Lisboa, Portugal; b IT - Instituto de Telecomunicações, 1049-001 Lisboa, Portugal; c Lisbon Unit for Learning and Intelligent Systems (LUMLIS), a unit of the European Laboratory for Learning and Intelligent Systems (ELLIS), 1049-001 Lisboa, Portugal

**Keywords:** Biomedical instrumentation, Biosignal acquisition, Biomedical devices, Do-it-Yourself (DiY)

## Abstract

Today, the use of biosignals is no longer limited to the traditional healthcare and medical domains, thanks to the application of biomedical engineering principles and devices in other domains, paving the way to the broader field of physiological computing. The increasing interest from the global engineering community, together with the challenges associated with the stringent requirements of biosignal acquisition, have motivated the development of enabling low-cost instruments for physiological sensing. Still, the use of some of these instruments in experimental activities and practical projects is still bounded by the cost and limited access to adequate support materials. In this paper, we present a novel low-cost hardware architecture especially designed for biosignal acquisition, and pre-programmed with a firmware optimized for real-time data acquisition and streaming. Our approach can be used seamlessly with available open-source software and APIs, without requiring extensive knowledge of electronics or programming. We also describe a series of tests conducted to evaluate the performance of this device, as a way of verifying its suitability for use in engineering and scientific work. Overall, the results presented here show that there is no loss of data in communication, accurate sampling rates, and high noise rejection capabilities in the tested conditions.

## Graphical abstract

**Figure:**
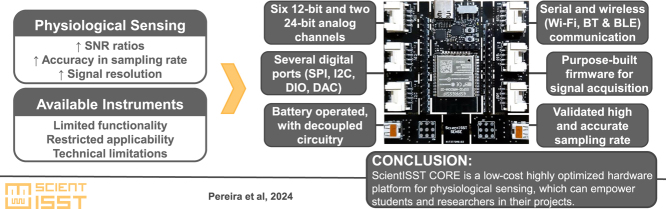

**Table:** 

**Specifications table**	

**Hardware name**	ScientISST CORE

**Subject area**	•*Educational tools and open source alternatives to existing infrastructure*

**Hardware type**	•*Other (low-cost hardware architecture designed for biosignal acquisition)*

**Closest commercial analog**	This hardware platform replaces Arduino boards in biosignal acquisition related tasks.

**Open source license**	Hardware - CERN Open Hardware License v1.1; Firmware - Apache License 2.0; Software API - MIT License.

**Cost of hardware**	74 EUR for 1 unit, 37 EUR for 1 unit without Grove connectors included.

**Source file repository**	OSF, http://doi.org/10.17605/OSF.IO/6RAB8

## 1 Hardware in context

**Fig. 1:**
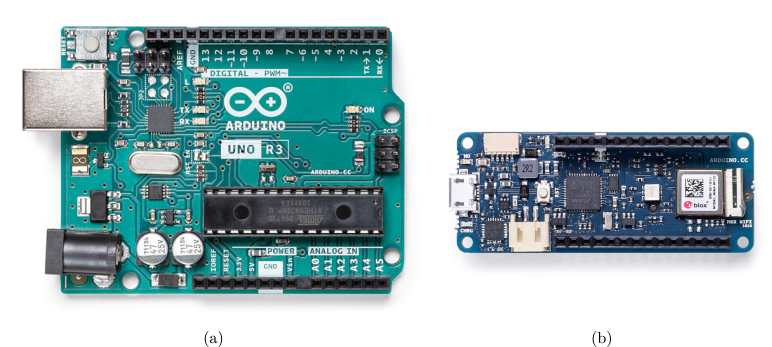
Example of two popular boards from the Arduino ecosystem: Arduino UNO (a) and Arduino MKR WiFi 1010 (b).

Over the past few decades, the development of low-cost hardware and open-source software has had a remarkable impact on several fields of expertise, including computer science, telematics, and electrical engineering. In particular, lowering the cost barrier and shortening the learning curve has resulted in the growth of the Do-it-Yourself (DiY) hardware community, which has in turn fostered the development of innovative interactive systems capable of sensing, processing, reacting, and interfacing the analog and digital worlds, in what is broadly defined as physical computing [1]. By far, the most renowned open-source electronics platform is the Arduino [2], due to the several advantages it offers over other systems, such as: a lower cost; simple and clear programming environment (Arduino IDE) that runs on Windows, MacOS, and Linux operating systems; and extensible open-source software and hardware. Over the years, numerous Arduino boards have been developed with distinct technical specifications, from the well-known Arduino UNO^1^ (Fig. 1(a)) to the more recent Arduino MKR WiFi 1010^2^ (Fig. 1(b)).

Meanwhile, biosignals have been extensively used in the healthcare and medical domains for over a century. However, they are becoming an increasingly popular topic of research within the global engineering community on account of the remarkable technical, methodological, and scientific achievements, brought on by the application of biomedical engineering principles and devices beyond the medical domain [3], [4]. Today, the potential applications of biosignals extend far beyond the classical medical domain into the broader nascent field of physiological computing, which includes: athletic performance studies [5], [6]; telemedicine and home care [7]; biosignal-controlled human–computer interfaces [8]; or biometric applications [9].

Physiological computing differs from physical computing in that the former focuses specifically on the use of biosignals. It is therefore associated with unique challenges related to the more complicated requirements of physiological data acquisition, such as the need for higher signal-to-noise ratios, greater accuracy in the sampling rate, and better signal resolution. As a result of these requirements, even the simplest biosignal acquisition tasks, in general, require prohibitively expensive hardware (often well above $10k). In recent years, efforts have been made to develop enabling low-cost instruments for physiological sensing, comparable to the DiY electronics prototyping platforms used in physical computing projects albeit with a modicum of success [10].

Although most Arduino-based toolkits are designed for physical computing applications [11], [12], providing little support for the development of projects involving biosignal acquisition, researchers have recently turned to the development of Arduino-compatible biosignal acquisition sensors, as is the case of the Pulse Sensor^3^ - a plug-and-play photoplethysmography (PPG) sensor for heart rate detection - or the MyoWare Muscle Sensor^4^ for electromyography (EMG) data acquisition.

Despite these efforts, Arduino-based approaches are still not entirely suitable for physiological computing applications. As mentioned before, biosignal acquisition has stringent requirements in terms of both sampling rate accuracy and tolerance to noise, which these platforms have proven not to be able to fulfill, due to high sampling rate drift, skew and jitter, as well as high sensitivity to noise, harmonic distortion and crosstalk between channels [13], [14]. Additionally, the technical skills needed to understand the software created for some biosignal acquisition sensors and correctly connect them to the Arduino board, pose a barrier to people looking to take their first steps in physiological computing, highlighting the need to make greater efforts towards improving the ease-of-use of such sensors.

**Fig. 2:**
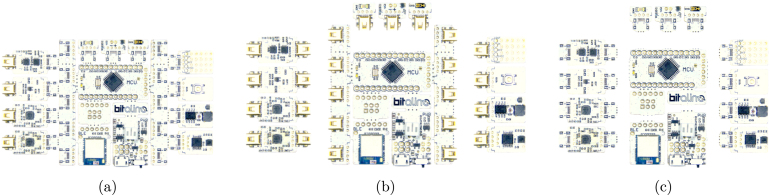
Different configurations of the BITalino biosignal acquisition hardware: Board (a); Plugged (b); and Freestyle (c).

Currently, the most analogous tool to the Arduino for applications involving biosignal acquisition is perhaps the BITalino [15], a low-cost biosignal acquisition system originally created in our research group, and that builds upon the guiding principles of existing physical computing hardware platforms. With its all-in-one hardware board format (Fig. 2(a)), it is designed to meet the learning and prototyping needs of any user interested in experimenting with its onboard physiological sensors. The BITalino also has two more configurations, namely: Plugged (Fig. 2(b)), which allows for flexible sensor combinations and facilitating experimentation with various setups, by having the analog front-end separated from the main board and connected via plugs; and Freestyle (Fig. 2(c)), which offers users total modularity to customize their setups as needed, by having individual blocks completely isolated from the main board. For overall characterization and additional information about BITalino, we refer the reader to [16].

Since its development, several studies have found that BITalino is a reliable tool for biosignal acquisition. Batista *et al.* [17] compared the quality of the data acquired by the BITalino (r)evolution and an older version of this system with a reference device, concluding that the signals obtained from both BITalino platforms were well correlated with the reference device. Additionally, Guerreiro *et al.* [14] demonstrated that even this older version of the BITalino outperformed Arduino platforms in terms of robustness to noise and sampling rate accuracy, due to the high skew and jitter caused by the considerable runtime overhead introduced by the Arduino libraries.

Despite these findings, the BITalino platform still exhibits some technical limitations. In particular, some specifications are not suitable for certain physiological computing applications, such as the maximum sampling rate of only 1000 Hz (e.g. acoustic signals in phonocardiography can require sampling rates upwards of 4000 Hz [18]) and the resolution of the analog input channels (BITalino only has 4 analog channels with 10-bit resolution and 2 with 6-bit). Additionally, BITalino does not support communication with external devices that use I2C or SPI protocols, and it is not compatible with the Arduino IDE, a programming environment nowadays first learned by most students, limiting its use in an educational environment.

Overall, the resources available in this area still face a number of limitations, in particular: limited functionality (e.g. devices only capable of measuring one or two pre-defined biosignal modalities, such as the OpenEEG Project [19]); lack of access to raw data, which limits applicability (e.g. the heart rate monitor in the MySignals platform^5^ only provides the post-processed cardiovascular signals); preclusion of interesting operations for educational and experimental purposes (e.g. direct control/interaction with the hardware) due to industrial property issues; or technical limitations for certain biosignal acquisition tasks.

**Fig. 3:**
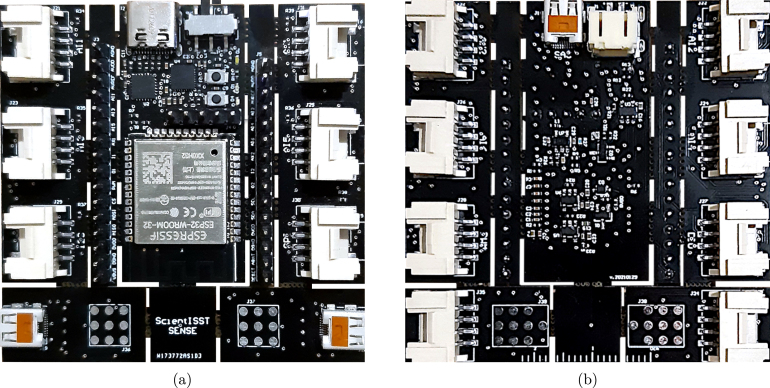
ScientISST CORE development board: top view (a) and bottom view (b).

In this work, we introduce ScientISST CORE^6^ (Fig. 3), a reference hardware design to accelerate experimentation in biomedical engineering which seeks to democratize the ability to rapidly prototype applications involving biosignal acquisition and processing, while also resolving some of the limitations found in similar low-cost platforms. Our goal is to provide the physiological computing community with a high-performance tool for developing innovative and creative engineering projects. It can be used in an out-of-the-box manner, due to the ecosystem of tools built around it, which includes sensors and several programming APIs. In the long term, we hope to dispel the preconceived notion many students, researchers, and practitioners have of biosignals as a complex and hard-to-reach topic due to lack of experience in this area.

The remainder of the paper is organized as follows: Section 2 characterizes the approach taken in developing the ScientISST CORE base hardware; Section 3 presents the hardware, firmware and software files needed to assemble and deploy this device; Section 4 contains the bill of materials; Section 5 and Section 6 present the build and operation instructions, respectively; and, finally, Section 7 describes the performance evaluation of the hardware, namely, quantitative tests performed to characterize the throughput, the temporal uncertainty (in terms of sampling rate accuracy), and the effects of battery discharge, along with example applications and the main conclusions.

## 2 Hardware description

In order to overcome the limitations described in Section 1, we devised ScientISST CORE, a novel low-cost hardware architecture designed with improved technical specifications for biosignal acquisition. Hereinafter, for the sake of simplicity, this device is referred to as CORE. The CORE board is built upon the ESP32-WROOM-32^7^ MCU module. At the core of this module is the ESP32-D0WDQ6^8^ chip, which includes two low-power Xtensa® 32-bit LX6 microprocessors, with a configurable CPU clock frequency from 80 MHz to 240 MHz. Additionally, this chip features 520 KB of SRAM and 4 MB of flash memory.

### 2.1 Analog interfaces

CORE exposes six main analog input ports to which any sensor with a compatible analog output can be connected using a Grove cable^9^ or a micro HDMI cable (depending on the connector soldered to the board). All analog inputs are connected to a signal conditioning circuit designed to bring the input voltage down to the measurement range of the internal Analog-to-Digital Converter (ADC) from the ESP32 module (ADC1, which has a range of 0–1.1 V). This ADC has a higher resolution (12-bit) when compared to the state-of-the-art, allowing for more precise measurements. Moreover, the ESP32 module integrates a second 12-bit internal ADC (ADC2); in CORE, one channel from this ADC is used to monitor the battery level, when using our purpose-built firmware.

Lastly, the CORE board integrates a high-resolution external ADC (24-bit effective resolution), more suitable for tasks that require a higher degree of precision in the conversion process. This ADC provides two additional analog input ports with the exact same behavior as the other six ports; in total, CORE exposes a total of 8 analog inputs.

### 2.2 Digital interfaces

A Serial Peripheral Interface (SPI) and an Inter-Integrated Circuit (I2C) ports are also included in the CORE board. These ports can be used to establish a communication line between the CORE board and external devices that use these types of digital interfaces. For instance, a common digital interface for memory card modules is SPI, while LCD displays and various sensors (e.g. distance, temperature, humidity sensors) commonly use I2C. While these ports may not be particularly useful for biosignal acquisition, as sensors designed for this purpose are mostly analog sensors, they enable the integration of other devices that can support the applications being developed.

Additionally, CORE exposes a pair of digital input and output pins (1-bit), useful for receiving synchronization signals from external devices or event annotation switches, as well as for sending synchronization signals to external devices or triggering external interfaces (e.g. a LED). A Digital-to-Analog Converter (DAC) port is also integrated to provide access to one of the two 8-bit DAC channels from the ESP32 module, which can be used to convert a digital signal into an analog voltage output.

At the bottom of the board, there are four breakout connectors (two Grove and two Female Micro HDMI) that are not connected to the rest of the board, but are included as breakout boards to facilitate the connection of external accessories, thus increasing its potential customization.

### 2.3 Power

CORE is battery operated, which means we can guarantee its independence from any high voltage power source during normal operation. However, this poses some problems that can affect measurements during biosignal acquisition. In particular, the voltage drop associated with the normal battery discharge and fluctuations in the power supply introduced by modules with high peak currents, as is the case of the aforementioned connectivity modules and digital integrated circuits [20].

In order to reduce the external supply voltage to the board’s operating voltage, and ensure a steady constant supply through all operational conditions, voltage regulators were incorporated into CORE. The board has two separated regulated 3.3 V outputs and grounds to independently power the analog and digital parts of the acquisition circuit. The reason behind this separation is that the digital circuitry can produce a considerable amount of noise in the power lines (e.g. when transmitting data). On the other hand, biosignal analog circuitry can be quite vulnerable to this noise, making this separation necessary to achieve maximum analog performance and prevent corruption from digital noise. Additionally, the board also has a regulated 1.65 V output to provide an offset voltage for biosignal sensors that have a bipolar measurement range (e.g. for voltage potential differential sensors used to acquire electrocardiogram (ECG), EMG, and related signals).

Additionally, CORE is fitted with a USB-C connector, which is used to recharge the battery, in addition to the functions detailed in Section 2.4. However, when a device is being powered by an external power source through the USB cable, it could be a potential hazard, if sensors that are coupled to a person’s body are connected as well. There is an increased risk of electric shock on account of the potentially high common-mode voltage on the USB signals and the low impedance path to the body via electrodes. To protect the user from this risk, several 0Ω resistors may be removed from the board in different combinations, as a way of providing distinct degrees of protection, by decoupling the analog circuitry from the USB power source. However, it is important to note that removing these resistors can limit the ability to reprogram the device or recharge the battery, so there is a trade-off between added protection to the user and some functionalities, as summarized in Table 1.

**Table 1:** Effect of resistor(s) configuration on ScientISST CORE’s operation.

		Effect on resistor(s) removal		
		Battery charge	Flashing possible	Analog circuitry decoupled from USB-5V
Resistor(s) to be removed	RVBUS RUSBP RUSBN	Not possible	Not possible	✓
	RLIPO	Only in OFF mode	Only in OFF mode	Not completely
	RVBD	✓	Only in ON mode if battery is plugged	Not completely
	RLIPO RVBD	Only in OFF mode	Not possible	✓
	None	✓	✓	Not completely

### 2.4 Communication

Apart from recharging the battery, the USB-C connector on the CORE board makes it possible to establish a serial communication line between the board and a PC for data transmission and reprogramming purposes. However, it is important to note that to flash new firmware onto the device, complete decoupling of the analog circuitry from the USB-5V lines is not possible (Table 1), so it may be necessary to shunt or solder a set of 0Ω resistors, which may have not been assembled in some boards to ensure complete decoupling.

For wireless data transfer, the CORE board supports three wireless communication protocols: Wi-Fi (using the 802.11 b/g/n wireless standard); Bluetooth v4.2; and Bluetooth Low Energy (BLE). By adopting both Wi-Fi and Bluetooth technology (classic and low energy), in combination with the battery-powered operation, we can ensure electrical isolation of the user from high voltage power sources, as well as broader interoperability when compared to other systems that only use one of these modules. For instance, using Wi-Fi enables direct connection to the Internet through a Wi-Fi router, as well as a wider physical range, whereas using BT or BLE requires connection of the system to a base station (e.g. computer or mobile phone). Alternatively, because the CORE board includes a SPI port, acquired data can be stored in a SD card when connected to this port using a memory card module. It is important to note that, when acquiring data to be stored to a SD card, only two modes are supported at the moment: all internal analog channels at 2000 Hz sampling rate; or all internal analog channels and 1 or 2 external analog channels at 100 Hz. After data acquisition, the data can then be retrieved by removing the SD Card, and using a provided Python script (Section 3.2) to convert the binary files to CSV files.

### 2.5 Firmware

By default, the CORE board already has a purpose-built firmware for physiological data acquisition, developed using the native ESP32 development framework, ESP-IDF.^10^ This firmware manages the behavior of the system in terms of, e.g., the sampling rate used and the sampled channels. To control the wireless data streaming (over Wi-Fi, BT, or BLE), record, and plot the data being received, the firmware supports two software APIs: the recommended ScientISST API (available natively in Python^11^), which has more features and is more actively maintained; and the deprecated BITalino API, which is only supported for backwards compatibility with the BITalino.

**Fig. 4:**
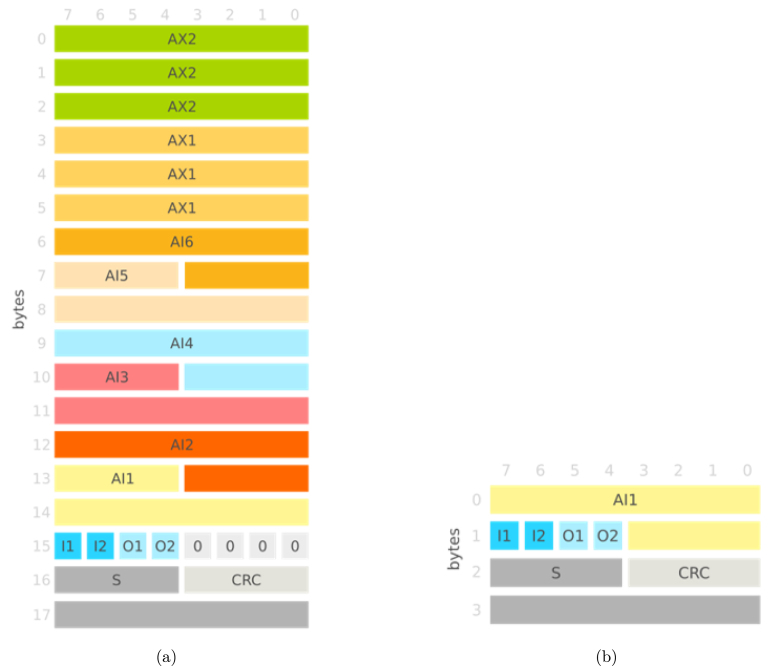
Example data packet structure for all analog channels (a) and just one internal analog channel (b) selected, namely: Sequence number (S); CRC code; Digital input channels (I1 & I2) and output channels (O1 & O2); and Analog channels associated with the internal ADC (AI1 to AI6) and to the external ADC (AX1 & AX2).

To transmit the data from the sampled input channels selected by the user, this data is first formatted into packets of variable size, ranging from 18 bytes (when all analog channels are selected) to 4 bytes (when only one 12-bit analog channel is selected). The data frame includes a 4-bit Cyclic Redundancy Check (CRC) code, generated using the CRC-4 algorithm [21], which enables frame data integrity check and allows for the detection and recovery from data packet inconsistency (e.g. caused by loss of packets in communication), as well as a 12-bit packet sequence number to identify cases of packet loss. Fig. 4 shows the structure of the data packets streamed by the CORE board.

**Fig. 5:**
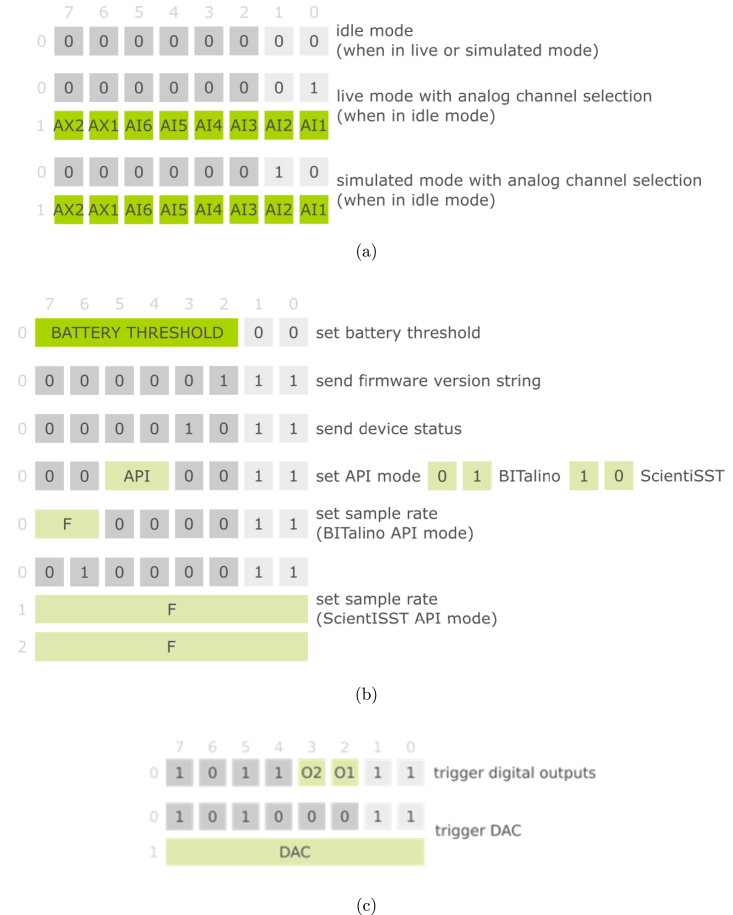
Commands recognized by the ScientISST CORE firmware, which includes: Operation commands (a); Configuration commands (b); and Trigger commands (c).

Although there are a number of software APIs available with high-level functions to control the operation of the board, as was mentioned before, the configurable settings can also be changed by sending 1, 2 or 3 byte commands from the base station directly to the CORE board. Fig. 5 shows the set of commands recognized by the purpose-built firmware.

Regarding the operation commands, CORE can operate under: “Idle Mode”, where the device is in stand-by and waiting to receive further commands; “Live Mode”, where data is being acquired in real time from the analog channels selected; and “Simulated Mode”, similar to the previous mode but in which the transmitted data is synthesized.

On the other hand, the configuration commands allow the user to modify certain settings related to the operation of the device, including the battery threshold, API mode (ScientISST CORE or BITalino), and sampling rate, as well as request information regarding the firmware version and device status. Furthermore, the trigger commands enable the use of the digital outputs and the DAC by sending the corresponding 1 or 2 byte command, respectively (Fig. 5).

Access to this low-level control of CORE expands its interoperability, by allowing the user to interface this device with other embedded systems. Additionally, it can be used in academic and pedagogical activities to explore data unpacking, CRC check, and other concepts related with digital communication using data acquired in real-time.

Unlike other devices found in the state-of-the-art, CORE is compatible with the Arduino IDE, meaning that custom firmware can be more easily created using this programming environment. Still, the Arduino framework, due to its cross-platform nature, produces firmwares that use more memory and are less performing when compared to the ones developed using the native ESP32 development framework. This negatively impacts the system’s sampling rate accuracy, hindering the use of the device in research-grade applications.

### 2.6 Summary

The main specifications of the CORE development board are summarized in Table 2. Furthermore, Table 3 summarizes some of these features comparatively to the hardware platforms found in the state-of-the-art designed specifically for physiological computing applications, highlighting how the former outperforms the majority of them. In light of these better specifications, the device we present is:

**Table 2:** ScientISST CORE specifications.

**MCU**	ESP32-D0WDQ6 with Xtensa® dual-core 32-bit LX6 microprocessor
**Sampling Rate (max.)**	1 kHz (all analog channels), 2 kHz (all external analog channels), 8 kHz (1 external analog channel), 10 kHz (1 internal analog channel)
**Analog Ports**	6 input (12-bit) ＋ 2 input (24-bit)
**Digital Ports**	2 input (1-bit) ＋ 2 output (1-bit) ＋ 1 output (8-bit) ＋ 1 I2C ＋ 1 SPI
**Data Link**	USB ＋ Serial ＋ I2C ＋ SPI ＋ BT ＋ BLE ＋ Wi-Fi
**Size**	6.3 × 6.3 × 1 cm
**Battery**	Rechargable 3.7 V LiPo battery
**Consumption**	∼46 mA in acquisition

**Table 3:** Comparison between ScientISST CORE and related work.

Platforms	Features						
	MCU				Analog input channels	Data link	Arduino IDE Compatibility
	Clock [MHz]	Flash memory	SRAM [kB]	Cores			
OpenEEG	16	16 kB	1	1	8 (10-bit)	Serial	No

MySignals HW	16	32 kB	2	1	6 (10-bit)	Serial ＋ Wi-Fi ＋ BLE	Yes

BITalino	8	32 kB	2	1	4 (10-bit) ＋ 2 (6-bit)	BT ＋ BLE ＋ Serial	No

ScientISST CORE	80–240	4 MB	520	2	6 (12-bit) ＋ 2 (24-bit)	USB ＋ Serial ＋ I2C ＋ SPI ＋ BT ＋ BLE ＋ Wi-Fi	Yes

• Fit for rapid prototyping in projects involving high performance biosignal acquisition and processing;
• Compatible with most off-the-shelve analog sensors and digital devices, as well as the Arduino IDE;
• Suitable for experiments in both education and scientific research settings, involving physiological sensing;
• Lower cost than most instruments for biosignal acquisition.

## 3 Design files

The design files, uploaded to and available at an OSF repository, consist of all the needed content to manufacture, assemble, and make the ScientISST CORE board fully operational (Table 4).

**Table 4:** List of design files available.

Design filename	File type	Open source license	Location of the file
scientisst-sense-hardware.zip	Markdown (.md), Excel (.xls), PDF (.pdf), KiCad (.kicad_sch, .kicad_pcb, .kicad_pro, kicad_mod), Gerber and 3D (.step, .stp) files	CERN Open Hardware License v1.1	http://doi.org/10.17605/OSF.IO/6RAB8

scientisst-sense-firmware.zip	Markdown (.md), C (.c, .h), Windows batch (.bat), shell script (.sh), and Python (.py) files	Apache License 2.0	http://doi.org/10.17605/OSF.IO/6RAB8

scientisst-sense-api-python.zip	Markdown (.md) and Python (.py) files	MIT License	http://doi.org/10.17605/OSF.IO/6RAB8

### 3.1 Hardware

**Fig. 6:**
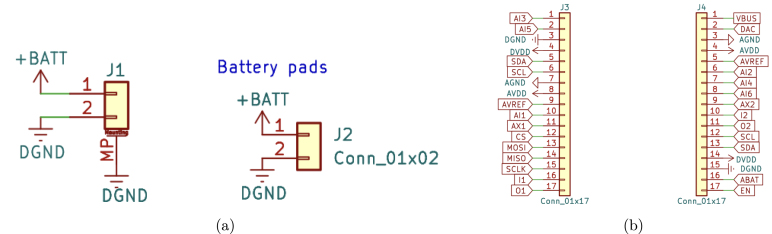
Schematic file ScientISST_Core.kicad_sch for the battery pads (a) and the pin headers (b).

**Fig. 7:**
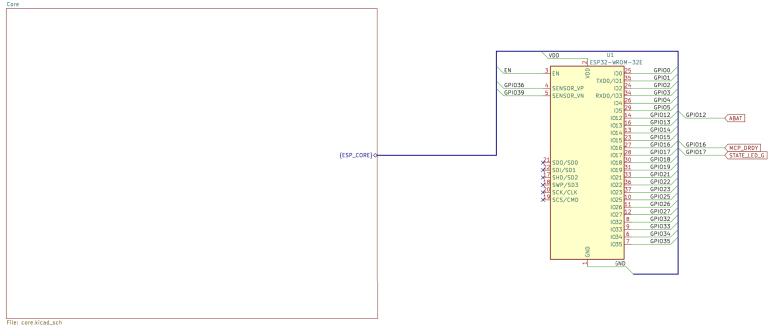
Schematic file ESP_WROOM-Core.kicad_sch.

**Fig. 8:**
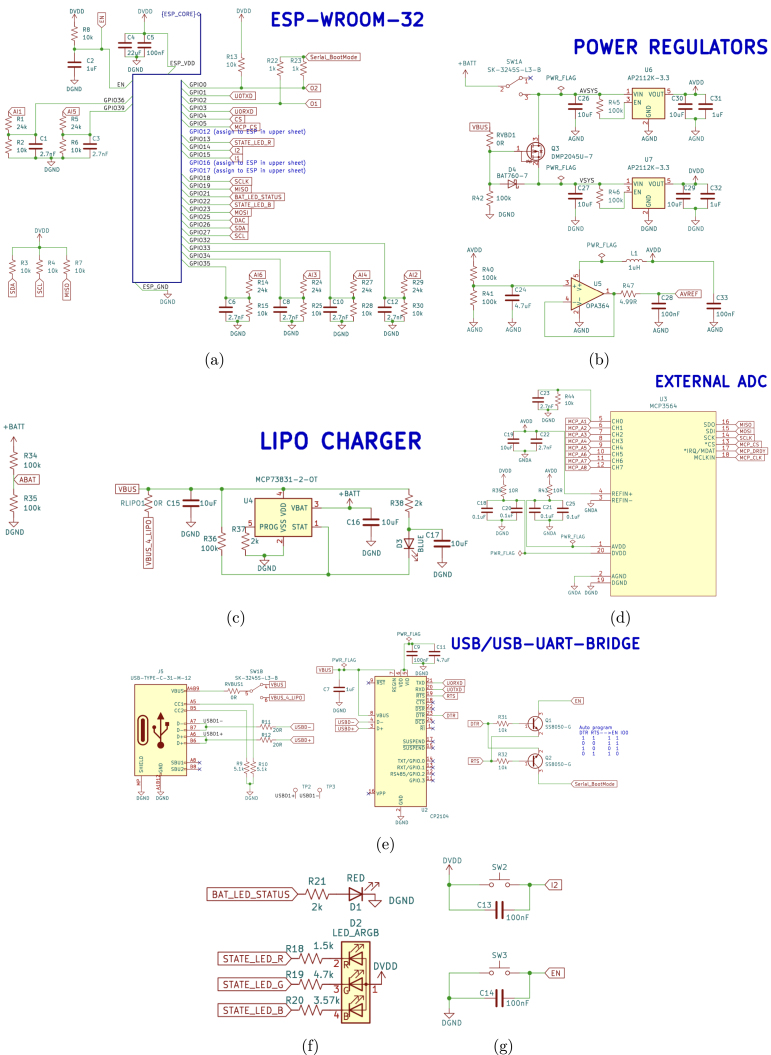
Schematic file core.kicad_sch for the ESP32-WROOM-32 MCU module (a), the voltage regulator circuit (b), the battery charging circuit (c), the external ADC (d), the USB connector and USB-to-UART bridge (e), the state LEDs (f), and the buttons (g).

**Fig. 9:**
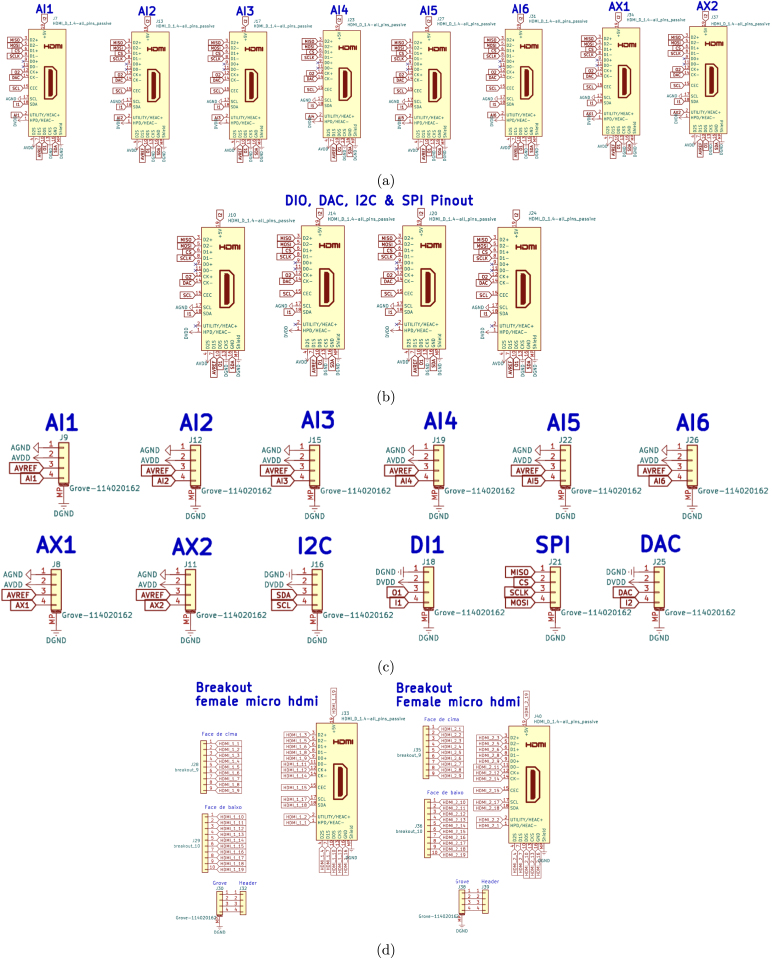
Schematic file connectors.kicad_sch for the HDMI connectors for the analog ports (a), the HDMI connectors for the digital ports (b), the Grove connectors (c), and the breakout connectors (d).

The .zip file named scientisst-sense-hardware is a folder that contains all the schematic, PCB design and 3D files of the ScientISST CORE board, which can be visualized with KiCAD.^12^ In this folder, there is also a Markdown file (titled README.md) with instructions on how to configure the library path in KiCAD to the folder libs/Scientisst_Core/, to open the files mentioned beforehand. Following this step, the Scientisst_Core folder contains the KiCAD project file (ScientISST_Core.kicad_pro). After opening this project, all the schematic files can be opened with the schematic editor, which include:

• **ScientISST_Core**: schematic of the battery pads and connectors for pin headers (Fig. 6).
• **ESP_WROOM-Core**: schematic of the ESP32-WROOM-32 MCU module (Fig. 7).
• **core**: schematic of the ESP32-WROOM-32 MCU module (continued), battery charging circuit, voltage regulators, external ADC, USB connector and USB-to-UART bridge, state LEDs and buttons (Fig. 8).
• **connectors**: schematic of the Grove/HDMI connectors for the six 12-bit analog channels (AI1 to AI6), two 24-bit analog channels (AX1, AX2), the pair of digital input and output pins (DIO), 8-bit DAC channel, SPI and I2C ports, as well as the four breakout connectors - two Grove and two Female Micro HDMI (Fig. 9).

**Fig. 10:**
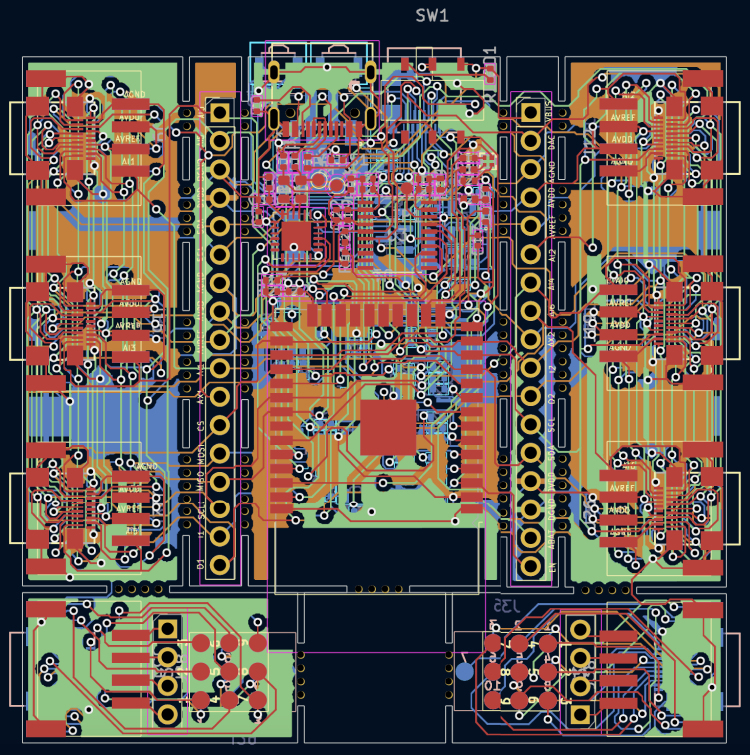
PCB design for the ScientISST CORE.

**Fig. 11:**
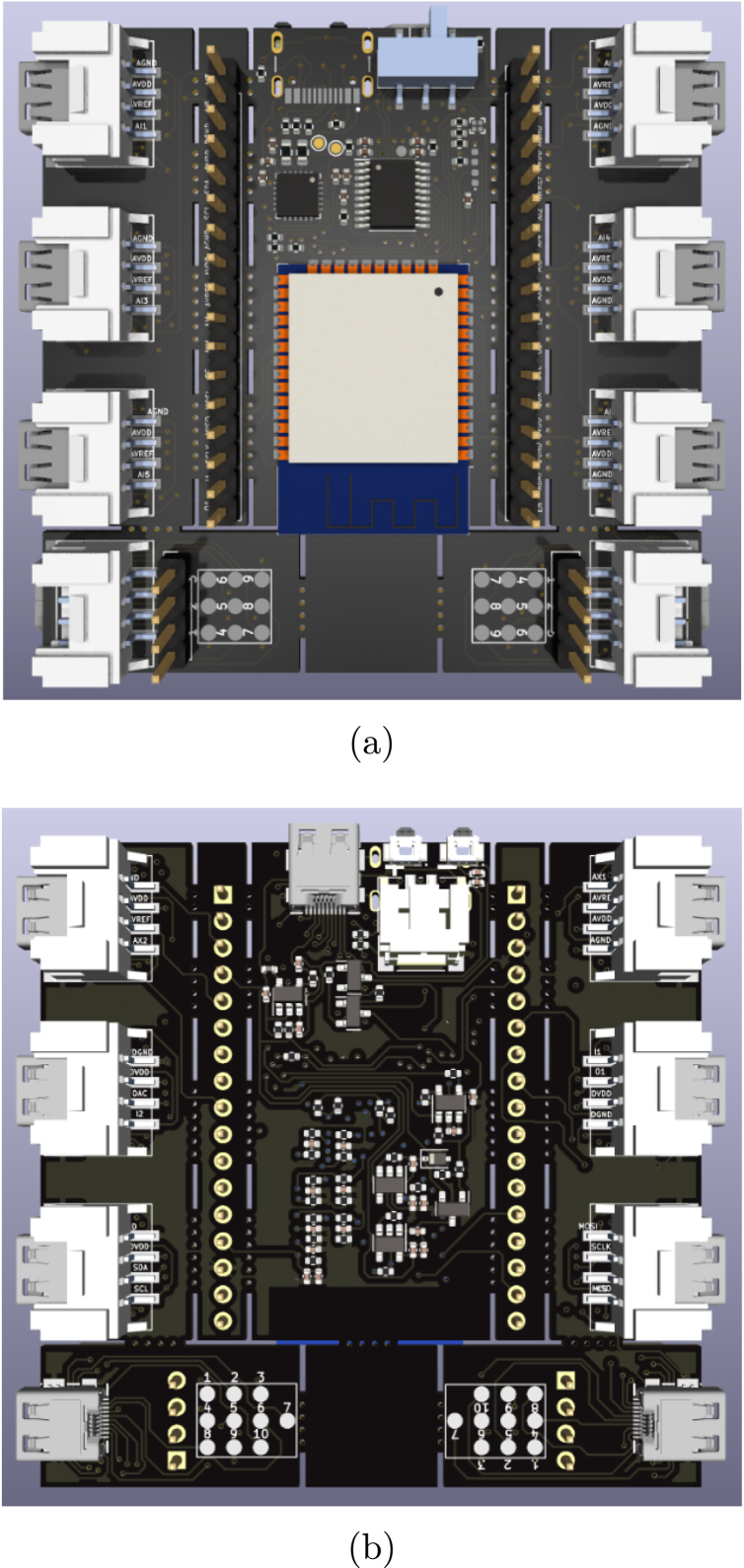
3D view of ScientISST CORE: Top view (a); and Bottom view (b).

All of the schematic files can be visualized also from a PDF file (ScientISST_Core.pdf), located inside the folder Scientisst_Core/docs. The Scientisst_Core folder also contains the PCB design file of the board (ScientISST_Core.kicad_pcb), which can be opened with the PCB editor (Fig. 10), which will automatically load the Gerber files for each image layer of the PCB design (found inside the folder Scientisst_Core/gerber). Furthermore, to view the PCB design in 3D, the PCB editor has an integrated 3D Viewer, which will load the .stp and .step files in the folder libs/Scientisst_Core/Scientisst_Core.3dmodels (Fig. 11).

Lastly, in the Scientisst_Core folder, there is an Excel spreadsheet (ScientISST_Core_BoM.xls) with all the components that make up the ScientISST CORE, which will be further detailed in Section 4.

### 3.2 Firmware

The .zip file named scientisst-sense-firmware is a folder that contains all the necessary files to install, configure, and flash the ScientISST CORE firmware onto the board. This firmware, which is based on the ESP-IDF framework, is responsible for initializing the device, configuring the sensors, and communicating with the various APIs. The firmware source files, which can be found in the main directory, are divided into tasks, with each task responsible for a specific function:

• **sci_scientisst.c/.h:** the main task, responsible for initializing the device, configuring the sensors, and creating the other tasks; terminates itself after the setup is complete.
• **sci_task_acquisition.c/.h:** responsible for acquiring data from the sensors and storing it in the buffers; a timer is used to wake this task up at the desired sampling rate, which can be changed at runtime using the API.
• **sci_task_battery_monitor.c/.h:** responsible for monitoring the battery level and turning on a red LED when the battery is low; does not interact with other tasks, and only runs when not using the Wi-Fi communication mode, because the device’s Wi-Fi driver uses the ADC2, so the latter cannot be used at the same time with this communication mode.
• **sci_task_com_tx.c/.h:** responsible for transmitting the data stored in the buffers to the API.
• **sci_task_com_rx.c/.h:** responsible for receiving and processing commands from the API, such as start/stop acquisition, change sampling rate, change API mode, active channels, etc.
• **sci_task_acquisition_sdcard.c/.h:** responsible for acquiring data from the sensors and storing it in the SD Card,^13^ when in this mode; this task is created instead of sci_task_acquisition, sci_task_com_tx and sci_task_com_rx.

As mentioned before, the firmware source files are not the only files that can be found in the scientisst-sense-firmware folder, but rather all the files necessary to install, configure and flash this firmware. As such, the firmware repository is structured as follows:

• **deps/:** external dependencies.
  – **BNO055_driver/:** Bosch Sensortec’s BNO055 driver.
  – **esp-idf/:** Espressif Systems’ ESP-IDF v4.4.4.
• **docs/:** documentation of the repository.
  – **doxygen/:** Doxygen generated documentation of the main/ directory.
  – **How_to_flash_scientisst/:** instructions on how to flash a CORE board.
  – **frames.jpg/.svg:** layout of the frames used to communicate between the device and the various APIs (Fig. 4, Fig. 5).
  – **SDCardBinaryFileFormat.md:** description of the binary file format used to store the data in the SD Card.
  – **StyleGuide.md:** style guide for the firmware.
• **main/:** firmware source files (all ScientISST files follow the format sci_XXXXX.c/.h).
  – **certs/:** cryptographic files used for secure communications protocols.
  – **communication/:** communication protocols.
  – **drivers/:** GPIO, WiFi, UART, timer.
  – **sensors/:** internal and external ADCs, BNO055.
  – **tasks/:** tasks created by the firmware.
  – **CMakeLists.txt:** CMake file.
  – **component.mk/**
  – **doxygen_config_file:** Doxygen configuration file.
  – **Kconfig:** allows configuring the firmware using the menuconfig tool (detailed in Section 5.2).
  – **main.c:** entry point of the ScientISST application.
  – **sci_macros.h:** global macros used by the firmware.
  – **sci_scientisst.c:** initialization of the device, task selection and creation, buffer allocation.
  – **sci_scientisst.h:** contains global variables.
  – **sci_version.h:** contains the version of the firmware, automatically updated with each commit.
• **SDCardFileConverter/:** Python script to convert the SD card’s binary files to CSV files.
• **www/:** files to generate the configuration HTML page.
• **CMakeLists.txt:** CMake file.
• **dependencies.lock:** ESP-IDF dependencies lock file.
• **get_idf.bat:** script to install and export to path the ESP-IDF for Windows.
• **get_idf.sh:** script to install and export to path the ESP-IDF for Linux/MacOS.
• **LICENSE:** Apache License 2.0.
• **Makefile**
• **partitions.csv:** custom ESP32 partitions.
• **README.md:** installation, configuration and flashing instructions of the firmware.
• **sdkconfig:** ESP32 ESP-IDF configurations.
• **sdkconfig.defaults:** ESP32 ESP-IDF configurations (defaults).
• **update_version.sh:** script to update the version of the firmware, used as a pre-commit hook to update sci_version.h with the latest commit hash.

### 3.3 Python API

The .zip file named scientisst-sense-api-python is a folder that contains all the necessary files to run the ScientISST CORE Python API. The Python program files, located in the folder scientisst, define classes and functions, that a user can use to establish communication and control the ScientISST CORE (e.g. start or stop data acquisition from specified channels, get the device’s current state), constituting this API’s main library.

Meanwhile, the sense_src folder contains auxiliary Python program files (for passing arguments, selecting available devices, saving data to a file, etc.), required to run the sense.py script. This script, which will be discussed further in Section 6, uses the functions and classes from both the API library and auxiliary files to simplify the connection and interaction with the ScientISST CORE for data acquisition and logging. In the API files, there is also the plot_output.py script, which, given the name of a data file acquired using sense.py, plots the data from each sampled channel.

The remaining files in the scientisst-sense-api-python include:

• **LICENSE:** MIT license.
• **README.md:** installation and usage instructions of the Python API.
• **requirements.txt:** external Python libraries required to run the Python API.
• **mkdocs.yml:** file to generate the documentation website,^14^ using the files located in the docs folder, where more information can be found on how to install and use this API, as well as detailed descriptions of its classes and functions.

## 4 Bill of materials

Table 5 contains the bill of materials for the ScientISST CORE board. Note that this table includes the unit pricing to purchase the necessary components to build a single board, but it is recommend to purchase these components, as well as the PCB, in larger batches at a smaller price, as these make building each device more cost-effective. In this case, the price for the PBC is derived from the cost to produce a batch of five boards (23.97 EUR).

## 5 Build instructions

### 5.1 ScientISST CORE PCB

The bare or assembled PCB for the ScientISST CORE board can be acquired by uploading the bill of materials (Table 5), as well as the schematics and PCB design files found in the scientisst-sense-hardware folder (Table 4) to any PCB assembler. However, it is important to note that most PCB assemblers charge a tooling setup cost to place components. Thus, it is recommended to order larger batches of assembled devices from PCB assemblers, to lower the overall cost per board. For a batch size of five boards, the assembly cost from PCBWay was estimated at 27.69 EUR, which is equivalent to approximately 5.54 EUR per board.

**Fig. 12:**
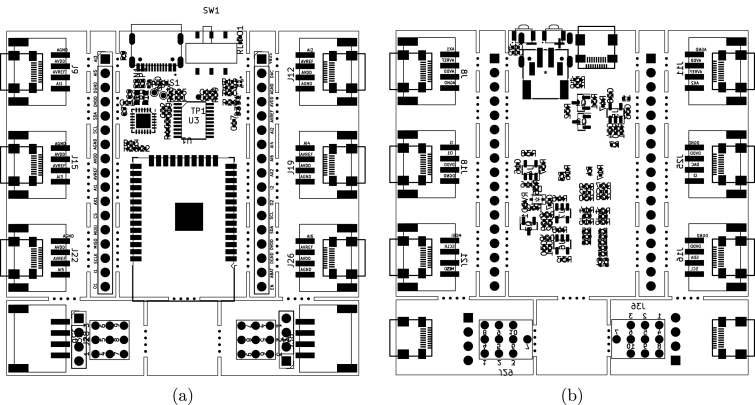
ScientISST CORE PCB layout: top view (a); and bottom view (b).

Another way to circumvent the high setup cost is the manual assembly of the PCB by soldering the components by hand in the bare PCB. This can be done using the following instructions:

1. Sort the components in the ScientISST CORE bill of materials (Table 5) into labeled component bins.
2. Open the ScientISST_Core.kicad_pcb file.
3. Toggle on only the silkscreen, mask and fabrication (fab) PCB image layers for the top of the board (F.), as depicted in Fig. 12(a).
4. Place the components with tweezers and solder them to their designator locations, which are displayed on Fig. 12 (due to the density of components, some designator locations are hard to decipher, so it is recommended to check which component belongs in each solder pad by clicking on it). Some of these components can be soldered using a soldering iron with a flux pen and solder, but for those smaller than the “0402 (1005 Metric)” size or that have smaller solder pads, such as U2, a heat gun or an oven should be used. When using an oven for manual assembly, solder paste and a stencil can be useful.
5. Repeat Steps 3 and 4 for the bottom of the board (B.), as depicted in Fig. 12(b).
6. Clean the assembled PCB with isopropyl alcohol to remove any excess flux.

It is important to note that manual assembly can be very challenging and time-consuming, requiring a lot of soldering experience, due to the large number of components, which oftentimes have a small footprint. Additionally, soldering irons operate with temperatures that can exceed 300 °C, and mishandling these equipment can cause severe burns or skin charring. As such, the most cost and time effective and safest way to acquire a CORE board will be through our website, which will become available in the near future for a fixed unit cost similar to the hardware and assembly cost mentioned before.

**Table 5:** ScientISST CORE bill of materials.

Designator	Component	Qty	Cost/unit (EUR)	Total cost (EUR)	Source of materials	Material type
C1, C3, C6, C8, C10, C12, C22, C23	Capacitor, SMD, 0402 (1005 Metric) package, 2.7 nF	8	0.09	0.72	Mouser Electronics	Multilayer Ceramic

C2, C7, C31, C32	Capacitor, SMD, 0402 (1005 Metric) package, 1 μF	4	0.09	0.36	Mouser Electronics	Multilayer Ceramic

C4	Capacitor, SMD, 0402 (1005 Metric) package, 22 μF	4	0.46	0.46	Mouser Electronics	Multilayer Ceramic

C5, C9, C13, C14, C18, C20, C21, C25, C28, C33	Capacitor, SMD, 0402 (1005 Metric) package, 100nF	10	0.034	0.34	Farnell	Multilayer Ceramic

C11, C24	Capacitor, SMD, 0402 (1005 Metric) package, 4.7 μF	2	0.09	0.18	Mouser Electronics	Multilayer Ceramic

C15, C16, C17, C19, C26, C27, C29, C30	Capacitor, SMD, 0402 (1005 Metric) package, 10 μF	8	0.09	0.72	Mouser Electronics	Multilayer Ceramic

D1	LED, SMD, 0402 (1005 Metric) package, Red	1	0.25	0.25	Mouser Electronics	AlInGaP/InGaN

D2	LED, SMD, 0404 (1010 Metric) package, RGB	1	0.41	0.41	Mouser Electronics	AlInGaP/InGaN

D3	LED, SMD, 0402 (1005 Metric) package, Blue	1	0.35	0.35	Mouser Electronics	AlInGaP/InGaN

D4	Schottky Diode, BAT760-7, SMD, SOD-323 package	1	0.37	0.37	Mouser Electronics	Silicon

J1	Connector, JST-S2B-PH-SM4-TBLFSN	1	0.518	0.518		Plastic and Metal

J5	Connector, USB-TYPE-C-31-M-12	1	0.2	0.2	LCSC Electronics	Plastic and Metal

J8, J9, J11, J12, J15, J16, J18, J19, J21, J22, J25, J26, J30, J38	Connector, Seeed Studio Accessories SMD Grove-114020164	14	2.6	36.4	Mouser Electronics	Plastic and Metal

L1	Inductor, SMD, 0402 (1005 Metric) package, 1 μH	1	0.09	0.09	Mouser Electronics	Ferrite

Q1, Q2	Bipolar Transistor NPN, SS8050-G, SMD, SOT-23 package	2	0.27	0.54	Mouser Electronics	Silicon

Q3	MOSFET Transistor, DMP2045U-7, SMD, SOT-23 package	1	0.36	0.36	Mouser Electronics	Silicon

R1, R5, R14, R24, R27, R29	Resistor, SMD, 0402 (1005 Metric) package, 24k	6	0.09	0.54	Mouser Electronics	Thick Film

R2, R3, R4, R6, R7, R8, R13, R15, R16, R17, R25, R26, R28, R30, R31, R32, R44	Resistor, SMD, 0402 (1005 Metric) package, 10k	17	0.11	1.87	Mouser Electronics	Thick Film

R9, R10	Resistor, SMD, 0402 (1005 Metric) package, 5.1k	2	0.009	0.018	Farnell	Thick Film

R11, R12	Resistor, SMD, 0402 (1005 Metric) package, 20R	2	0.09	0.18	Mouser Electronics	Thick Film

R18	Resistor, SMD, 0402 (1005 Metric) package, 1.5k	1	0.09	0.09	Mouser Electronics	Thick Film

R19	Resistor, SMD, 0402 (1005 Metric) package, 4.7k	1	0.09	0.09	Mouser Electronics	Thick Film

R20	Resistor, SMD, 0402 (1005 Metric) package, 3.57k	1	0.09	0.09	Mouser Electronics	Thick Film

R21, R37, R38	Resistor, SMD, 0402 (1005 Metric) package, 2k	3	0.09	0.27	Mouser Electronics	Thick Film

R22, R23	Resistor, SMD, 0402 (1005 Metric) package, 1k	2	0.09	0.18	Mouser Electronics	Thick Film

R33, RLIPO1, RUSBN1, RUSBP1, RVBD1, RVBUS1	Resistor, SMD, 0402 (1005 Metric) package, 0R	6	0.09	0.54	Mouser Electronics	Thick Film

R34, R35, R36, R40, R41, R42, R45, R46	Resistor, SMD, 0402 (1005 Metric) package, 100k	8	0.09	0.72	Mouser Electronics	Thick Film

R39, R43	Resistor, SMD, 0402 (1005 Metric) package, 10R	2	0.09	0.18	Mouser Electronics	Thick Film

R47	Resistor, SMD, 0402 (1005 Metric) package, 4.99R	1	0.09	0.09	Mouser Electronics	Thick Film

SW1	Switch, SK-3245S-L3-B	1	0.52	0.52	LCSC Electronics	Plastic and Metal

SW2, SW3	Tactil Switch, B3U-3000P	2	0.88	1.76	Mouser Electronics	Plastic and Metal

U1	ESP32-WROM-32E, WiFi Modules (802.11) SMD module, 4 MB SPI flash, PCB antenna	1	2.47	2.47	Digi-Key	Semiconductor

U2	CP2104-F03-GM, Interface Bridges, USB to UART, 3 V, 3.6 V, QFN, 24 Pins, −40 °C	1	2.7	2.7	RS Amidata	Semiconductor

U3	MCP3564R-E/ST, Analog to Digital Converters - ADC 24-bit delta-sigma ADC w/Vref, Quad channel, 3 V	1	5.81	5.81	Mouser Electronics	Semiconductor

U4	MCP73831T-2ACI/OT, Battery Charger for 1 Cell of Li-Ion, Li-Pol battery, 6 V input, 4.2 V/500 mA charge, SOT-23-5	1	0.7	0.7	Digi-Key	Semiconductor

U5	OPA364AIDBVR, Operational Amplifiers - Op Amps 1.8 V High CMR RRIO Op Amp	1	1.79	1.79	Mouser Electronics	Semiconductor

U6, U7	AP2112K-3.3TRG1, LDO Voltage Regulators 600 mA CMOS LDO 50 mA 3.3 V 250 mV	2	0.33	0.66	Mouser Electronics	Semiconductor

PCB	ScientISST CORE PCB	1	4.794	4.794	PCBWay	PCB

### 5.2 Flashing the ScientISST firmware

In order to flash the firmware onto the CORE board, the first step is to install the ESP-IDF prerequisites on a computer, following Espressif’s guide^15^ for that computer’s operating system (Windows, MacOS, or Linux). The next step is to download the firmware repository from either OSF or Github. From the latter, the source code archives can be either downloaded from the repository view or by cloning the repository using Git from the command line with the following command:

**Figure:**
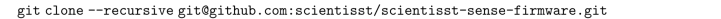

After this step, move the command terminal to the newly created folder named scientisst-sense-firmware, in order to install and load Xtensa’s toolchain using the get_idf.bat script for Windows users, or the get_idf.sh for MacOS or Linux users. To install, which only needs to be done once, the following command must be run in the terminal:

**Figure:**
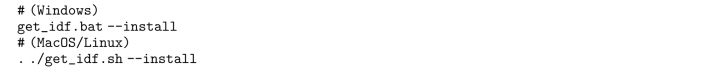

Then, in order to load Xtensa’s tools, which must be done every time a new terminal is opened, run the same command used to install but without the --install option.

With the installation complete, the next step is to configure the firmware using the menuconfig tool. To open this tool, run the command:

**Figure:**
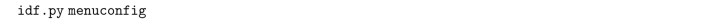

**Fig. 13:**
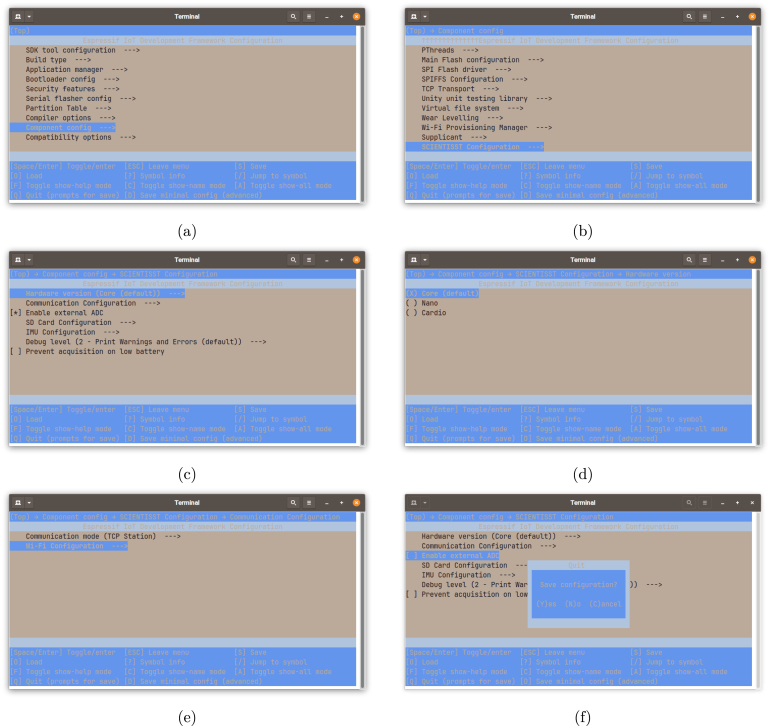
Espressif IoT Development Framework configuration menu layout for each configuration step: Top page (a); Component config (b); ScientISST configuration (c); Hardware version (d); Communication configuration (e); and Save configuration (f).

This command will open the Espressif IoT Development Framework configuration menu, shown in Fig. 13(a). In this menu, navigate to the “Component config” option (Fig. 13(b)), and from there go to the last option, “ScientISST configuration” (Fig. 13(c)), in order to configure the desired options. These include:

• **“Hardware version”:** where the user can select the hardware version/board model, which, in this case, will be the default CORE (Fig. 13(d));
• **“Communication configuration”:** gives the option to select the communication mode, which is set to BT by default, but when Wi-Fi is selected, a Wi-Fi configuration menu will be available so that the user can input the Wi-Fi SSID and password, host IP and port (Fig. 13(e));
• **“Enable external ADC”:** selecting this option enables data acquisition from the two external analog ports;
• **“SD Card configuration”:** where the SD card mode can be enabled (if this is enabled and a SD card is present, the device will always run in this mode, otherwise it will run in BT mode) and the number of external ADC channels can be selected (external ADC must be enabled beforehand);
• **“IMU configuration”:** gives the option to enable the IMU and select the type of data to acquire (Euler angles, angular velocity and/or linear acceleration);
• Option to prevent data acquisition when the battery is low.

It is recommend for the user not to make further changes in the Espressif IoT Development Framework Configuration menu, apart from the options mentioned before (unless they are intimately familiar with the board’s hardware), as this tool does not verify if the physical hardware supports the selected options. When the user has configured the CORE firmware according to their needs, the “S” key needs to be pressed to save the configuration (Fig. 13(f)).

Finally, to flash the configured firmware onto the CORE board, this device must be first connected to the computer using the USB-C port with an appropriate cable. Afterwards, to begin the flashing procedure, load Xtensa’s tools again and run the idf.py script, using the following commands (depending on the computer’s operating system):

**Figure:**
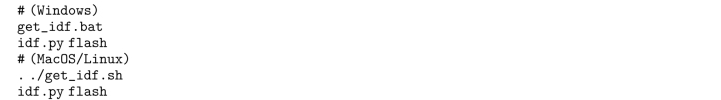

**Fig. 14:**
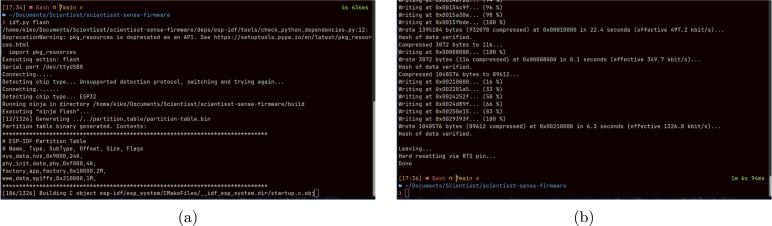
Displayed messages in the terminal when flashing the firmware in a Linux operating system, after running the command (a) and after successfully flashing (b).

After running the idf.py flash command, the flashing procedure will begin, as shown in Fig. 14(a), but, bear in mind that this process can take a few minutes. When the board has been flashed successfully, the command terminal will display a similar message to the one depicted in Fig. 14(b). If, however, an error occurs at any step of the installation, configuration or flashing of the firmware, it is recommended to consult the trobleshooting section in the README.md file of the firmware repository, or request assistance by creating an issue in the GitHub repository.

## 6 Operation instructions

In this section, instructions will be provided on how to install and use solely the ScientISST Python API to perform data acquisition and streaming, as this API is the one currently being maintained, and, consequently, possesses more features compared to the BITalino API.

Similarly to the firmware, the Python API repository can be either downloaded repository from OSF, or from Github by downloading the source code archives from the repository view or by cloning the repository using Git from the command line with the following command:

**Figure:**
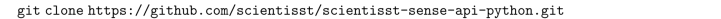

Alternatively, this repository can also be installed with Python’s package installer (pip), using the following command from the command line:

**Figure:**
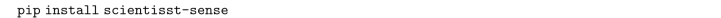

The next step is to install the dependencies required to run the Python API (if not already installed). These include five packages: pyserial (essential), pylsl (required to stream data using Lab Streaming Layer protocol, which will be detailed later), pydbus (required for Linux users to automatically get pairable devices in BT mode), numpy, and matplotlib (only necessary if the data is to be plotted after acquisition). In the command line, these packages can be installed individually using the pip install <package> command, or more simply by using the requirements.txt file in the API repository:

**Figure:**
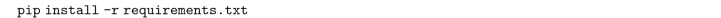

With the API and its dependencies installed on the computer, it should now be possible to interact with the CORE board and connected sensors (if flashed with our purpose-built firmware). To simplify this interaction, the API repository contains a script, sense.py, that, when run from the command line, connects to the CORE device and begins data acquisition, providing the option to store the received data in a CSV file. This script also allows the selection of channels to be sampled, the sampling rate, and the duration of data acquisition from the command line, along with implementing file data saving in the background (using a different thread). In addition, it provides the option to stream the acquired data via Lab Streaming Layer (LSL), a protocol that manages networking, time synchronization, real-time access, and, optionally, centralized data collecting, display, and disk recording for the unified collection of measurement time series in research trials.

To use this script, after moving the command terminal to the API directory, simply use the following command:

**Figure:**
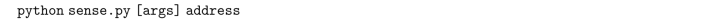

Where [args] are the optional arguments, which will be detailed later, and address is the only positional argument, corresponding to the address of the CORE board we wish to connect with. When using TCP/UDP communication, this is the server port (for all operating systems), whereas, when using BT communication, it is the BT serial COM port (for Windows users), the serial port address (for MacOS users), or the BT MAC address (for Linux users).

It is important to note that the address argument is also not required to run the sense.py script in BT mode, as long as the user has paired the device with the computer before running the script, which will then automatically display a list on the command line with all the paired ScientISST devices found and their addresses to select from:

**Figure:**
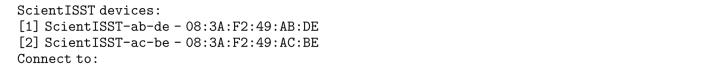

For manual selection of the device’s address, we direct the reader to the “Manual Selection” section in our “Getting Started” guide, found in this repository’s documentation website, where instructions on how to pair a device and find its address are available for each operating system.

Regarding the optional arguments of the sense.py script, the list is as follows:

• -h, --help: shows a help message, with the usage, description, and arguments of sense.py.
• -f FS, --frequency FS: allows the selection of the sampling frequency (FS, default: 1000 Hz).
• -c CHANNELS, --channels CHANNELS: allows the selection of the analog channels to sample data from, where 1 to 6 corresponds to the internal channels, while 7 and 8 to the first and second external channels (AX1 and AX2), respectively (CHANNELS, default: 1,2,3,4,5,6).
• -d DURATION, --duration DURATION: allows the selection of the duration of data acquisition, in seconds (DURATION, default: unlimited). If left unlimited, data acquisition can be stopped by hitting CTRL-C.
• -o OUTPUT, --output OUTPUT: allows acquired data to be recorded to an output CSV file (OUTPUT, default: None).
• -r, --raw: when this argument is present, data is not converted from its raw format to mV.
• -s, --lsl: when this argument is present, data is streamed using the LSL protocol. Use the command python -m pylsl.examples.ReceiveAndPlot to view the data stream.
• --script SCRIPT: when this argument is present, the received data frames are sent to the script with the given path (SCRIPT), that inherits the CustomScript class and runs custom code every time the sense.py script reads data from the device.
• -q, --quiet: disables printing of the received data frames in the command line.
• -v, --version: shows the sense.py script version.
• --verbose: enables the logging of sent/received bytes.
• -m MODE, --mode MODE: allows the selection of the communication mode. Currently supported modes: bt_classic, tcp, tcp_ap (MODE, default: bt_classic).

Using the arguments detailed beforehand, interaction with the CORE board is highly simplified. If, for example, a MacOS user wanted to acquire data from the analog channel AI1 at a 10 Hz sampling rate, and save the data to a CSV file with the name “output”, after pairing a CORE board, it would only be necessary to run the following command:

**Figure:**
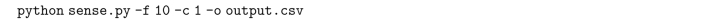

**Fig. 15:**
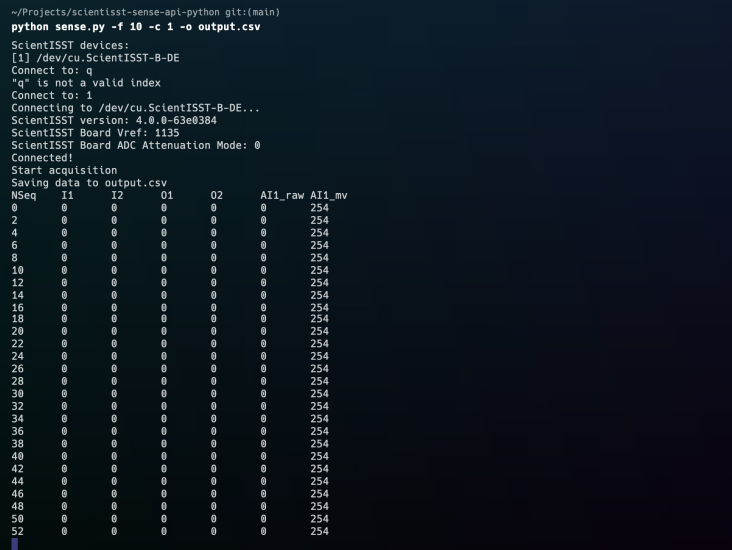
Data acquisition from the analog channel AI1 at a 10 Hz sampling rate, in a MacOS operating system.

After this command is run and the device address is selected, connection between the PC and device is established, with several details regarding the firmware and hardware being printed onto the terminal, including the firmware version, the board’s reference voltage (in mV) and ADC attenuation mode, as depicted in Fig. 15. As data acquisition is being carried out, the sequence number (NSeq), the values from the digital input (I) and output (O) ports, as well as the raw values from the selected analog port (in this case, AI1), expressed in both quantization levels (raw data) and millivolts, are also printed onto the terminal, allowing for a cursory examination of the acquisition in terms of data loss and integrity.

For more examples on the correct usage of each of these positional arguments, we again refer the reader to the “Examples” section in our “Getting Started” guide, found in this repository’s documentation website.

However, before starting data acquisition, it is important to, first, make sure the device is turned ON, and has sufficient battery (when the battery is low, a red LED will be turned on, if the device is not using the Wi-Fi communication mode). If everything is well, the state LED on the board should be blinking white before pairing, and stop blinking and remain white after pairing. The next step is to ensure any sensor or device connected to the board’s analog or digital interfaces is done so correctly (it is important to recall the operating voltage of 3.3 V), and that the board is unplugged from any high voltage power source, especially if any sensor is coupled directly to a person’s body. After these steps have been taken, everything should be ready for data acquisition using the sense.py script, which, if run successfully, will be signaled by rapid blinking of the white state LED on the board.

After data acquisition has been completed, the repository also contains a Python script to plot the acquired data from each selected channel (plot_output.py). To do so, in the command line, simply use the following command:

**Figure:**
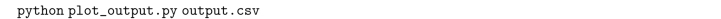

which will load the given output file (output.csv, in this case) and plot the channel data, as shown in Fig. 16.

**Fig. 16:**
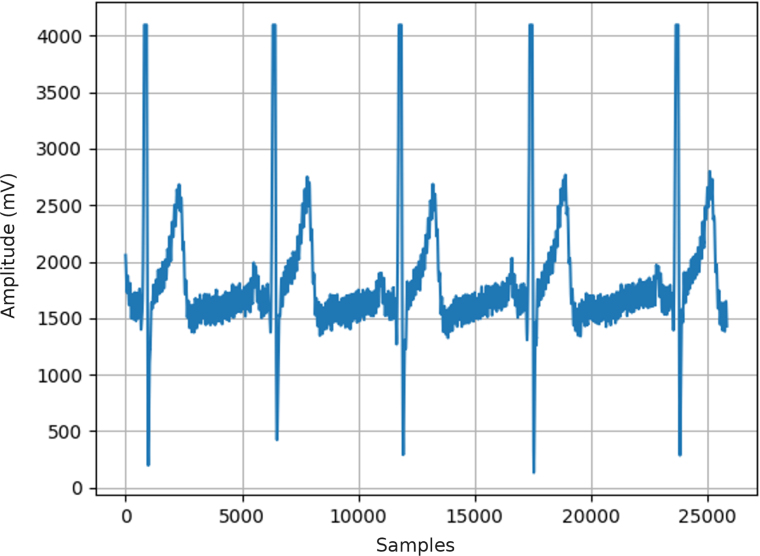
Example plot of ECG data acquired with a sensor connected to the analog channel AI3.

While the sense.py script makes interaction with the CORE device much simpler, the user is also free to create their own custom Python scripts using this API’s classes and functions (detailed in the API Reference), to make this interaction more suitable to their educational, academic, or scientific needs.

## 7 Validation and characterization

To ensure that the requirements described in Section 1 were fulfilled, tests were performed to evaluate the performance of the CORE board, namely, concerning the temporal accuracy of the ADC process (i.e. sampling rate accuracy), throughput, and effect of battery discharge. For these experimental tests, signals were synthesized using either the Tektronix AFG3102 Function Generator^16^ or the CORE board itself, depending on the tests being carried out.

### 7.1 Clock cycle characterization

While a device’s maximum sampling rate can be an important selection criterion for signal acquisition, a higher achievable sampling rate is not the only indicator of good performance when it comes to biosignal acquisition hardware. Another important aspect to take into consideration when developing these devices is the need for high accuracy in the sampling rate, as many biosignal-related applications are time sensitive and, consequently, do not tolerate large deviations in the sampling rate.

For this reason, tests were performed to characterize the clock cycle of the CORE board by having one of its General-Purpose Input/Output (GPIO) pins oscillating with different interrupt periods, namely 100, 1, 0.25 and 0.125 ms. This process was controlled by a timer set up with half of the interrupt period to invert the digital output in the pin, generating a square wave, which was acquired using an oscilloscope (Tektronix MDO3054).^17^ Table 6 displays the measured wave period statistics extracted for each interrupt period tested, which characterize the clock cycle, and subsequently the accuracy of the sampling rate up to 16 kHz. The number of cycles acquired varies in order to have one million samples for each interrupt period tested. However, some cycles had to be discarded, due to a limitation in the oscilloscope that sporadically would not record all the data, hence altering the measured period in the affected cycle.

The results from Table 6 show that both the mean and median of the measured wave periods coincide with the interrupt period, for all values tested. Furthermore, the standard deviation of the measured period, as well as the maximum, and minimum deviation are all very small, although the values for these statistics are slightly higher for smaller values of the interrupt period (0.25 and 0.125 ms), which was expected since the lower the interrupt period value is the more prominent the deviations from this value become. Even so, the small deviations exhibited in the measured period demonstrate the accuracy of the clock cycle in the ScientISST CORE, which should translate into precise sampling rates up to 16 kHz during biosignal acquisition. In reality, the maximum achievable sampling rate will not reach this value, as it is also dependent on the amount of data transmitted, among other factors, which will be discussed in Sections 7.2, 7.3, 7.4.

**Table 6:** Clock cycle characterization results.

Interrupt period/# Cycles	Measured period statistics				
	Mean (ms)	Median (ms)	Standard deviation (ms)	Max deviation (ms)	Min deviation (ms)
100 ms/3999	100	100	$1.81\times 10^{-11}$	$3.41\times 10^{-11}$	$1.39\times 10^{-14}$
1 ms/40000	1	1	$1.88\times 10^{-12}$	$4.77\times 10^{-12}$	0
0.25 ms/79999	0.25	0.25	$9.99\times 10^{-3}$	$1\times 10^{-2}$	$9.99\times 10^{-3}$
0.125 ms/80128	0.125	0.125	$5.01\times 10^{-4}$	$6.48\times 10^{-3}$	$4.8\times 10^{-4}$

Overall, the main reason for these clock cycle characterization results is the fact that the CORE has a much better-performing microcontroller unit (MCU) than other devices in the state-of-the-art. This is further reinforced by CoreMark,^18^ a simple albeit sophisticated benchmark provided by the EDN Embedded Microprocessor Benchmark Consortium (EEMBC), which is designed specifically to test the functionality of processor cores by producing a single-number score that allows users to make quick comparisons between the performance of different processors. In fact, the processor cores used in the ESP32 module have a CoreMark score of 924.26, whereas ATMega microcontrollers (most commonly found in devices from the state-of-the-art) at most have a score of 20.91, indicating, at minimum, an almost 42× improvement from the state-of-the-art in terms of MCU performance.

### 7.2 Waveform period measurement

To further characterize the temporal accuracy of the ADC process, square waves with 10 or 100 Hz frequency, synthesized by the Tektronix AFG3102 Function Generator, were acquired using the CORE board at different sampling rates. First, this acquisition was done using only one 12-bit analog channel from the internal ADC, followed by using one and then two of 24-bit analog channels from the external ADC, up to the maximum sampling rate achievable without data acquisition becoming unreliable, due to errors. Additionally, some of the more demanding internal and/or external ADC channel configurations, in terms of transferred data volume, were also tested for the 100 Hz square wave.

**Table 7:**
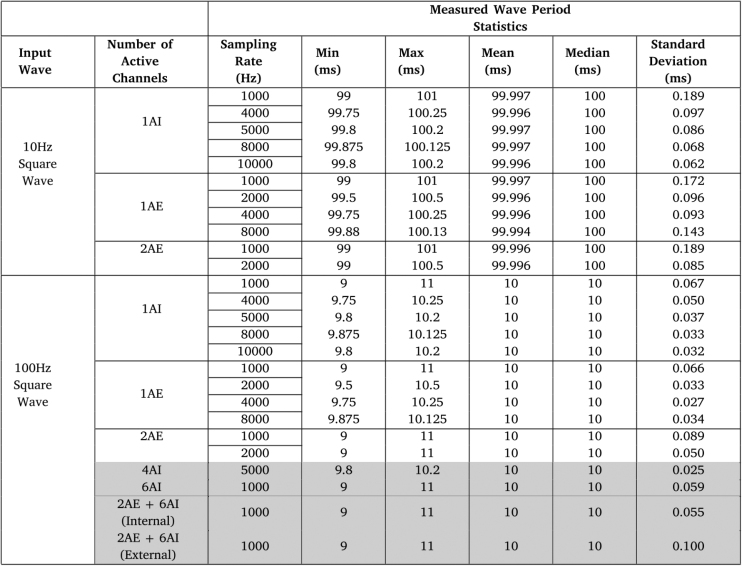
Waveform period measurement results for the 10 Hz and 100 Hz square waves, using different ADC channel configurations.

For all tested ADC channel configurations, data was acquired from the selected channels for 240 s, corresponding to 2400 cycles for the 10 Hz square wave, and 24000 cycles for the 100 Hz square wave. From the obtained signals, we extracted some statistics from the measured wave period values, namely their mean, median, standard deviation, maximum, and minimum. Table 7 shows the measured wave period statistics results obtained for each sampling rate, waveform tested, and tested channels, with the most demanding configurations mentioned before highlighted in gray.

The results displayed in Table 7 show that, while using one 12-bit internal analog channel, for all the sampling rate values tested below or equal to 10 kHz, the mean and median values of the measured waveform period approximately coincide with the expected values for both the 10 Hz and the 100 Hz square waves (100 ms and 10 ms, respectively). Additionally, the standard deviation of the measured wave period is low (in the order of magnitude of $10^{-2}\;\mathrm{ms}$ for most tested sampling rate values).

For the cases where higher standard deviations are observed, this is reflected in the higher maximum and lower minimum wave period values obtained, which did not seem to have a large impact on the mean and median values obtained. In fact, for every sampling rate value tested, the range of measured wave period values obtained is always centered around the anticipated value, from which can be deduced that these values oscillate closely to the expected value.

Similar results were also obtained for both the 10 Hz and 100 Hz square waves acquired using one or two 24-bit external analog channel, for all the sampling rate values tested, with the mean and median values of the measured waveform period again approximately coinciding with the expected values, and small standard deviation values.

Furthermore, while the maximum achievable sampling rate when using an internal ADC channel is 10 kHz, it decreases to 8 kHz when an external ADC channel is used instead of an internal ADC channel, and to 2 kHz when two external ADC channels are used instead. This is again to be expected, taking into account the increased size of the data packets being transmitted (6/9 bytes with one/two external ADC channel(s), compared to 4 bytes with one internal ADC channel), but it is not the sole reason for this decrease. In fact, when using the external ADC at high sampling rates (especially when using multiple channels), this ADC starts to respond with invalid results. Thus, the communication speed must be reduced to prevent this and stabilize the ADC process, which ultimately limits the maximum achievable sampling rate.

In spite of that, as was previously mentioned, when using one or two external ADC channels, the maximum sampling rate achieved was 8 kHz and 2 kHz, marking a 8× and 2× improvement compared to similar devices in the state-of-the-art, respectively, broadening this device’s uses to applications that require both signal sampling at a higher rate and a more precise conversion process from the analog to the digital domain.

For the most extreme use cases highlighted in gray, again the maximum achievable sampling rate decreases with the number of ADC channels used, for the same reasons stated before. However, from the similar results of the measured wave period statistics obtained, it can be concluded that using more channels does not significantly change the behavior observed in the acquired signals.

Nevertheless, in the cases where every single analog channel (i.e., two 24-bit and six 12-bit analog channels) or every single internal analog channel (i.e., six 12-bit analog channels) are being used, the maximum sampling rate achieved was 1 kHz, which is still on par with similar devices in the state-of-the-art. But, it is important to note that this represents only the most extreme use cases (most applications will not require the use of all these channels), and the level of precision in the ADC provided by the use of the 24-bit analog channels is not necessary for most biosignal acquisition tasks.

Altogether, the results from Table 7 indicate that using sampling rates of up to 10 kHz with one 12-bit analog channel guarantees a reliable and accurate conversion of the acquired signal from the analog to the digital domain, but the acquisition process is unable to comply with the sampling period when using a sampling rate above this value, which will be explored further in Section 7.3. Furthermore, the results from the configurations involving the external ADC indicate that, while using the 24-bit analog channels requires a trade-off between communication speed and stability in the ADC process, the external channels can still be used for tasks requiring a more precise conversion, with a sampling rate of up to 8 or 2 kHz when using only one or two external channels, or up to 1 kHz when adding on top of that all six internal channels.

**Table 8:**
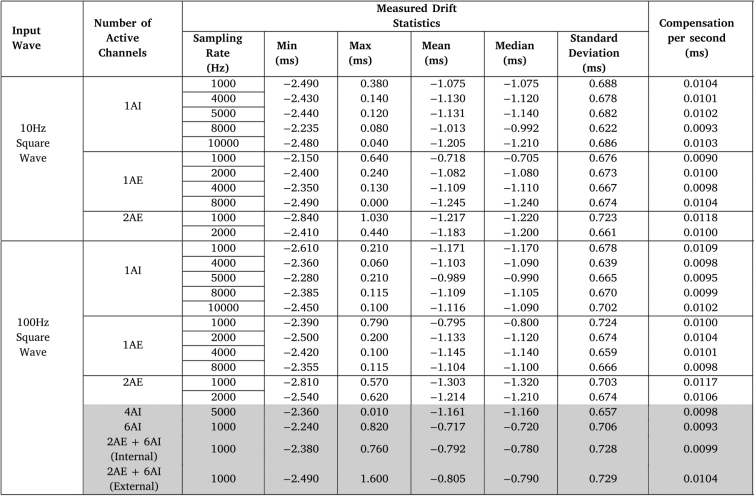
Drift characterization results for the 10 Hz and 100 Hz square waves, using different ADC channel configurations.

### 7.3 Drift characterization

Over time, temporal inaccuracies in the ADC process may accumulate, leading to errors in the acquisition of biosignals, namely in the measured signal period. To characterize this drift, the square waves acquired by the ScientISST CORE, whose period was analyzed in Section 7.2, using several configurations of ADC channels, were simultaneously and synchronously recorded with an oscilloscope. The drift was then obtained by computing the value of the difference between the time instants when a rising edge occurred in the wave acquired by the CORE board, comparatively to when this same edge occurred in the oscilloscope. The statistics extracted from the acquired values for each sampling rate, waveform frequency value and analog channels tested are displayed in Table 8, together with the compensation per second, which will be explained later.

From the results shown in Table 8, it can be observed that, regardless of the tested conditions, the values from each measured drift statistic extracted are similar between each other, with no particular channel configuration or sampling rate value tested deviating from the rest. The only statistic that shows more variance than the rest is the maximum drift, which appears to decrease as the sampling rate increases for the same channel configuration. However, this positive drift largely only occurs when the rising edge of the square wave occurs in the time interval between two sample points, which is why this value decreases with the increase in sampling rate, as this leads to a decrease in that interval.

**Fig. 17:**
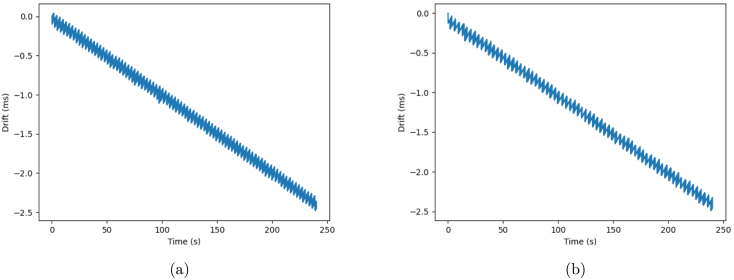
Example plots of the approximate linear progression of the drift over time, for the 10 Hz square wave, using a 12-bit internal analog channel at 10 kHz (a), and a 24-bit external analog channel at 8 kHz (b).

Additionally, it can be deduced that, for the most part, the drift is negative, due to the larger absolute values of the minimum compared to the maximum, as well as the negative mean and median values. This is confirmed by the plots of the measured drift over time, depicted in Fig. 17, Fig. 17.

In the available APIs, it is assumed that each data packet is spaced by 1/SR (where SR is the sampling rate), when, in reality, they are spaced by (1/SR+ϵ), with this error ϵ in seconds. In other words, because the “real” sampling period is greater than it should be theoretically, over time the wave acquired by the CORE board progressively falls behind the same wave acquired by the oscilloscope, becoming out of phase with it. The error (ϵ) between the “real” and theoretical sampling period can be explained by a variety of factors, namely inaccuracies in the chip’s timers, calculation processing times, and/or delays in certain processes that are running simultaneously.

However, the overall results are quite positive, and these temporal discrepancies in the ADC process are not a reason for concern for a number of reasons. First, in an acquisition lasting 240 s, the largest absolute value of drift observed is only 2.840 ms (using one external ADC channel at 1 kHz), meaning that while these temporal inaccuracies in the ADC process accumulate, they do so very slowly, which limits their impact in the acquisition of biosignals. In fact, as was concluded in Section 7.2, the results from the wave period measurement indicated a reliable and accurate conversion of the acquired signal from the analog to the digital domain. Secondly, by observing the plots of the measured drift over time for each condition tested (Fig. 17, Fig. 17), we were able to conclude that the drift can be approximated by a linear function, given by the following equation:

(1) $$f\left(x\right)=\frac{Min}{240}\times x$$

where x is the current time (in seconds) and $\frac{Min}{240}$ is the slope of the function, given by the quotient of the minimum drift value registered in Table 8 for that specific sampling rate and channel configuration, and the duration of data acquisition (240 s, in this case). The absolute value of the slope for each tested condition is also displayed in Table 8, under the “Compensation per second (ms)” column, which translates to the expected absolute value of drift (in ms) accumulated per second. As such, should the need arise to regularize the timestamps and correct the drift in post-processing, the absolute value obtained from f(x) at a certain time instant (x) can be added to that timestamp.

It is important to note that, although this correction will reduce the effect of drift in signal acquisition, it is not perfect, because, as was previously mentioned, the evolution of drift over time is not a perfect linear function. In fact, it can be observed from the example plots in Fig. 17, Fig. 17 that there is a sawtooth behavior in the drift, arising from the fact that, in the acquisition APIs, the timestamps are deduced from the selected sampling rate, when in reality temporal inaccuracies in the ADC process can exist, as was explained before, leading to some sampled wave periods having more or less samples than expected. However, these deviations are so small that they have very little impact in the integrity and reliability of CORE’s data acquisition.

Lastly, regarding the maximum achievable sampling rate, it is noteworthy to highlight that the sampling rate values tested are all factors of 80 MHz (i.e. 80000 kHz). In fact, it was found that the drift in the ADC process can only be compensated properly, when the quotient of 80 MHz and the selected sampling rate is an integer. The reason behind this relationship between the drift and the sampling rate is the base clock frequency of the ESP32 MCU module (80 MHz); when working with sampling rate values that do not divide this base frequency by a whole number of cycles, the timer for analog channel sampling, which works at this same base frequency, has to resort to approximations that introduce greater amounts of drift.

In sum, from the drift characterization obtained with different ADC channel configurations and sampling rates, it can be concluded that the CORE board is capable of accurate and reliable conversion of signals from the analog to the digital domain, as long as the ratio between the base clock frequency and the selected sampling rate is an integer, exhibiting minimal drift in these conditions, which can be compensated in post-processing.

### 7.4 Throughput analysis

The throughput of the CORE board refers to how much data can be transferred in a reliable manner from this device to a base station in a given amount of time. Thus, the throughput is limited by both the number of analog channels being sampled and the sampling rate used, as these influence the amount of transmitted data per unit of time. The more channels being sampled, the larger the size of the transmitted data packets, and the higher the sampling rate, the more data packets are sent per unit of time. As a result, there is a trade-off between the number of analog channels sampled and the maximum achievable sampling rate, as was seen in Sections 7.2, 7.3. In order to analyze this trade-off, data acquisition was performed using one to six 12-bit and/or one to two 24-bit analog channels simultaneously, with an incremental increase of the sampling rate between acquisitions, up until the point data acquisition became unreliable, due to errors, or timeouts began to occur, meaning the device was no longer able to transmit the amount of data required. Table 9 summarizes the results of this analysis.

Using only the internal channels, the lowest maximum sampling rate (3 kHz) was obtained when five or all six 12-bit analog channels were being sampled, corresponding to a data packet size of 10 or 12 bytes, respectively. Even so, this is a marked improvement comparatively to similar devices in the state-of-the-art, which only sport a maximum sampling rate of 1 kHz for all available channels [16]. Furthermore, it can be observed from Table 9 that, as less internal analog channels were selected, the maximum achievable sampling rate increased, as was expected. In particular, for a single analog channel, corresponding to a data packet size of 4 bytes, the maximum sampling rate was 10 kHz, more than 3.3× higher than the one achieved by the same device with six 12-bit analog channels, and 10× higher than other devices in the state-of-the-art.

**Table 9:** Throughput analysis results, obtained using a combination of AI (analog internal) and/or AE (analog external) channels.

# Analog Channels/# Bytes	Sampling rate				
	1 kHz	3 kHz	5 kHz	8 kHz	10 kHz
	Timeouts				
2 AEs ＋ 6 AIs/18B		8s			
2 AEs ＋ 5 AIs/16B		8s			
2 AEs ＋ 4 AIs/15B	–		8s		
2 AEs ＋ 3 AIs/13B	–		8s		
2 AEs ＋ 2 AIs/12B	–		8s		
2 AEs ＋ 1 AIs/10B	–		8s		
2 AEs/9B	–		8s		

1 AEs ＋ 6 AIs/15B	–		8s		
1 AEs ＋ 5 AIs/13B	–		8s		
1 AEs ＋ 4 AIs/12B	–		8s		
1 AEs ＋ 3 AIs/10B	–	–		8s	
1 AEs ＋ 2 AIs/9B	–	–	–		8s
1 AEs ＋ 1 AIs/7B	–	–	–	–	
1 AEs/6B	–	–	–	–	

6 AIs/12B	–		8s		
5 AIs/10B	–		8s		
4 AIs/9B	–	–		8s	
3 AIs/7B	–	–		8s	
2 AIs/6B	–	–	–	–	
1 AIs/4B	–	–	–	–	

On the other hand, when using a single external 24-bit ADC channel, the maximum sampling rate achieved was 10 kHz, the same value achieved with one or two internal 12-bit channels, which was to be expected when taking into consideration that using one external channel or two internal channels leads to data packets of the same size (5 bytes), due to the external ADC’s increased resolution (24-bit vs. 12-bit).

When using a single external ADC channel with an increasing number of internal ADC channels, the maximum sampling rate again decreases from 10 kHz to 8 kHz, when two internal channels are also used, from 8 kHz to 5 kHz, when three are used, and from 5 kHz to 3 kHz, when four to six are used. Similarly, when sampling two external ADC channels and zero to four internal ADC channels, the maximum sampling rate remains the same (3 kHz). Only in the most extreme cases in terms of data packet size, when all analog channels (17 bytes) or all except for a single internal ADC channel (15 bytes), does the maximum sampling rate lower to 1 kHz. As such, even in these cases, this value only comes to show that the CORE board is still on par in terms of performance with other devices in the state-of-the-art.

Through this analysis, we were able to demonstrate the ScientISST CORE’s ability to transmit the acquired data at markedly higher sampling rates than the state-of-the-art in the majority of available ADC channel configurations, broadening its uses to applications that require signals to be sampled at a higher rate, which could not be met by other low-cost biosignal acquisition hardware (e.g., phonocardiography in which the sampling rate used is typically 4 kHz or above) [18].

### 7.5 Battery discharge analysis

**Fig. 18:**
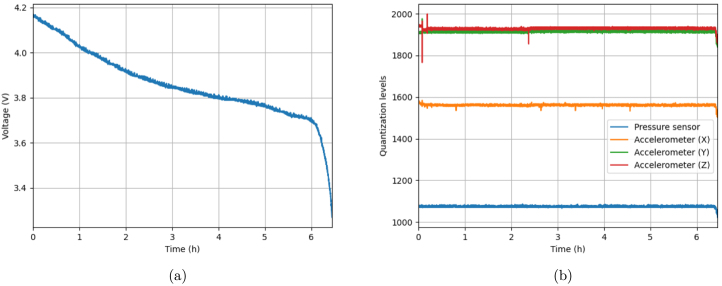
Battery discharge effect in the ADC process, when the CORE is sampling four 12-bit analog channels at a 1 Hz sampling rate, connected to an air pressure sensor and an accelerometer, illustrated through the battery discharge curve depicting the drop in the battery voltage level over time (a), and the graph of the air pressure sensor and accelerometer readings in quantization levels (b).

In order to analyze the effect of battery discharge on signal acquisition, a test was conducted using a fully charged Lithium-Ion/Lithium-Polymer battery with 3.7 V - 900 mAh and setting the device to sample not only the voltage level of the battery, but also four of the six 12-bit analog channels at a 1 Hz sampling rate. The four channels were connected to an air pressure sensor and the X, Y and Z channel outputs of an accelerometer. The battery discharge curve and the graph of the sensor readings obtained from this test are displayed in Fig. 18(a) and Fig. 18(b), respectively.

**Fig. 19:**
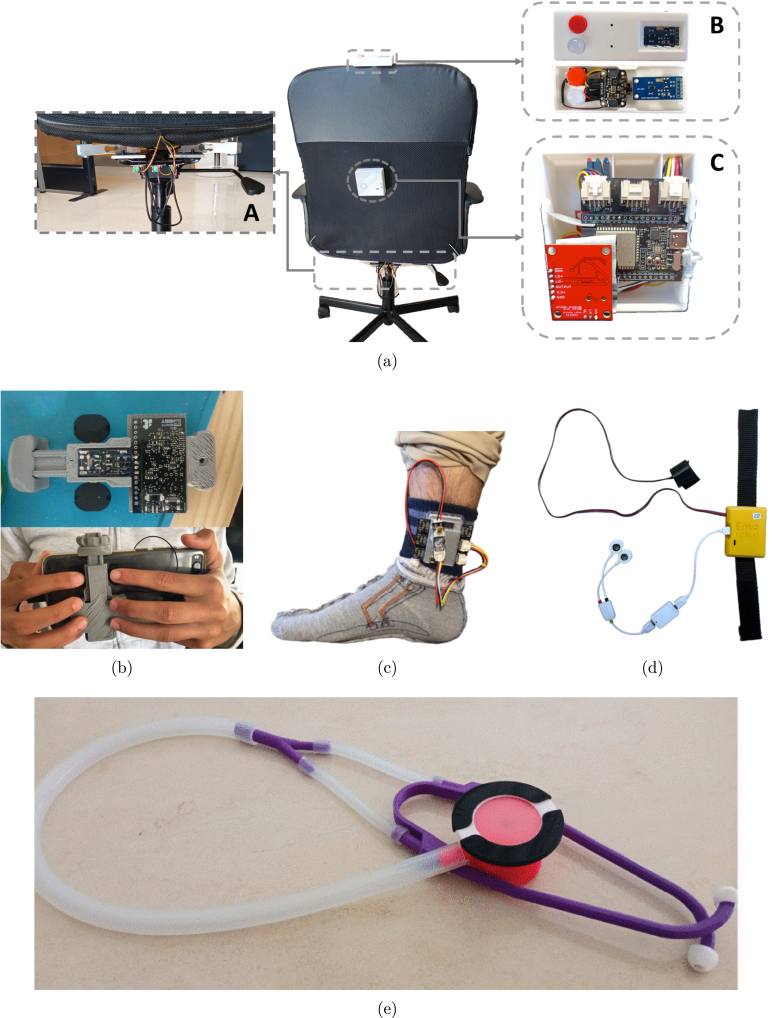
Examples of prototypes developed with the ScientISST CORE board: Smart chair system (a), with main units labeled as follows - (A) straight bar load cells and respective ADCs, (B) environmental sensors, status LED and configuration button, (C) ScientISST CORE, ECG sensor connected to armrests, and multiplexer [22]; Smartphone cover prototype (b), illustrating its use [23]; Prototyped proof-of-concept wearable smart sock (c) [24]; Wrist-worn device (d) with electrodermal activity (EDA) and PPG sensors [25]; Electronic stethoscope (e) for phonocardiography and ECG monitoring [26].

From the battery discharge curve, it can be concluded that, under the aforementioned conditions, the CORE board has an autonomy of approximately 6.5 h in continuous data acquisition in “Live Mode”. However, it is important to note that this autonomy will vary depending on the amount and type of sensors plugged to the device, as well as on the sampling rate selected.

Since the sensors were left for the most part undisturbed during the course of this test, the readings obtained should be mostly constant, showing little to no variation. Other than some sporadic spikes in the accelerometer values from disturbances in the environment, Fig. 18(b) shows exactly this behavior, which indicates that the board’s operating voltage remains steady throughout acquisition, even though the battery voltage level is dropping, thanks to the voltage regulators incorporated. Only when this level drops below 3.4 to 3.3 V an impact on the acquisition process is observed. This is evidenced by the sharp drop in all sensor readings, before the device shuts down, as expected, on account of the fact that the voltage regulators require an input voltage level above 3.3 V to maintain the 3.3 V output voltage, necessary for the correct operation of the MCU and, consequently, of its ADCs.

### 7.6 Example applications and deployments

As a reference hardware designed to accelerate experimentation and prototyping in applications involving biosignal acquisition and processing, the ScientISST CORE board has better technical specifications than other similar low-cost platforms for biosignal acquisition, as shown in Sections 2, 7, making it suitable for a greater number of applications in this area.

**Fig. 20:**
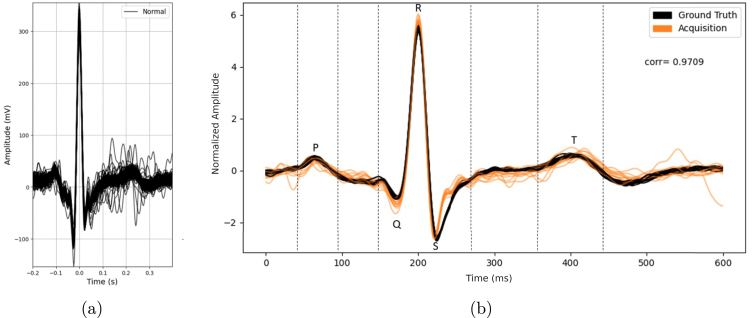
Examples of the data output from the prototypes developed with the ScientISST CORE board: Overlapped ECG signals acquired from the armrests of a smart chair system (a) [22]; and Overlapped ECG signals acquired from a smartphone cover prototype and ground truth signals (b) [23].

**Fig. 21:**
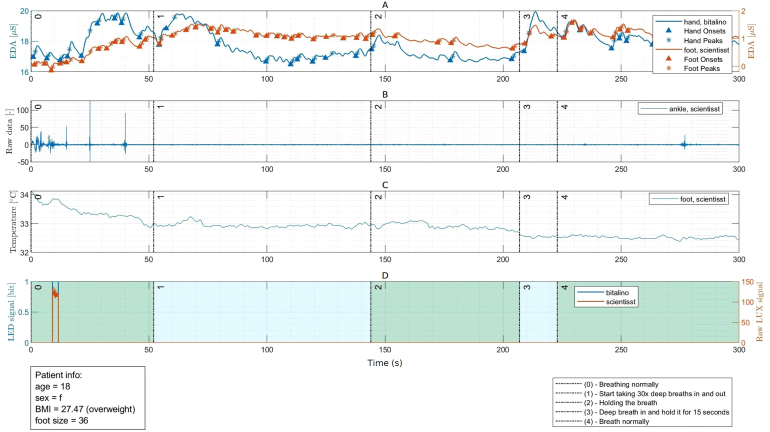
Validation of foot-based EDA measurement using the smart sock prototype in a single subject. The EDA signals were simultaneously acquired from the hand (using pre-gelled electrodes and the BITalino (r)evolution Plugged device) and the foot (via the smart sock). Hand signals (blue) served as the reference, while foot signals (orange) were analyzed as the experimental setup. Onsets and peaks of EDA signals are marked. Data were recorded during the Wim Hof Breathing Method (WHBM), a breathing technique known to activate the sympathetic nervous system. The plots illustrate: (A) EDA signals (top); (B) Foot acceleration (body movement); (C) Foot temperature; and (D) Synchronization signals (LED and light sensor/LUX), which confirm optical synchronization between devices. The WHBM session phases are segmented and numbered (0–4), highlighting: (0) Baseline breathing; (1) Deep breathing; (2) Breath-holding; (3) Breath-holding with strain; and (4) Recovery breathing [24].

**Fig. 22:**
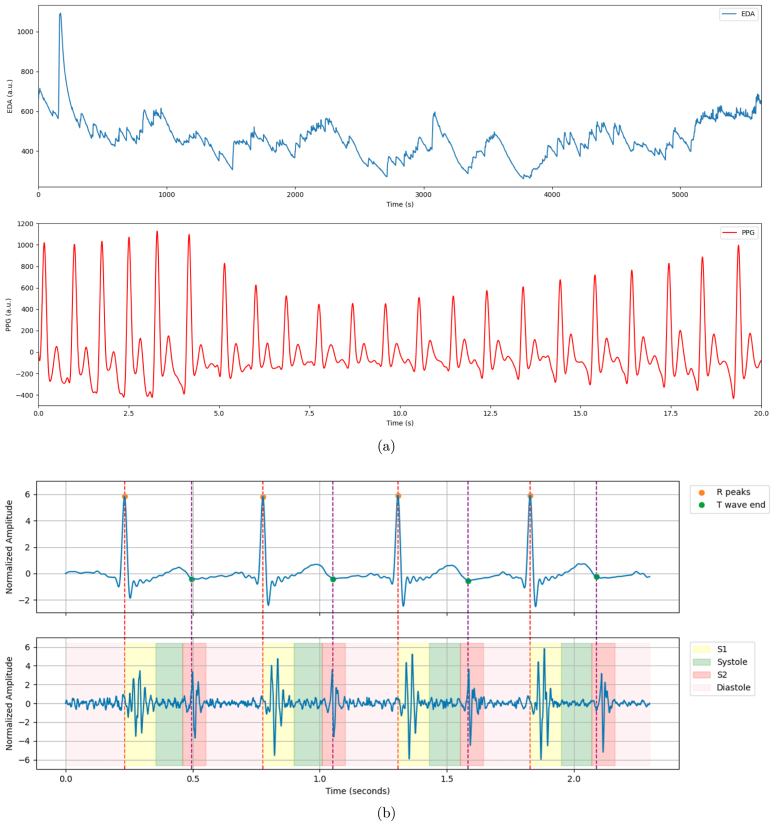
Examples of the data output from the prototypes developed with the ScientISST CORE board (continued): EDA and PPG signals acquired from the wrist-worn device (a) for emotion recognition during a movie session [25]; ECG and PCG signals acquired with the electronic stethoscope (b), where the PCG is segmented into the fundamental heart sound states, which are aligned with the R-peak and end T-wave positions of the ECG [26].

At the time of writing, the CORE board has been deployed in various projects, in both the educational and academic settings, with some of these leading to a number of published papers. These include the development of: a smart chair system for posture classification, using load cells, environmental and invisible ECG monitoring, using conductive fabric electrodes on the armrests (Fig. 19, Fig. 20) [22]; a prototype smartphone cover with a built-in ECG sensor, using conductive polymeric electrodes (Fig. 19, Fig. 20) [23]; a wearable smart sock with integrated accelerometer, temperature, PPG and EDA sensors, using textile electrodes (Fig. 19, Fig. 21) [24]; a wrist-worn device that collects physiological data from built-in PPG and EDA sensors for emotion recognition (Fig. 19, Fig. 22) [25]; and an electronic stethoscope adapted from a previously validated 3D-printed acoustic stethoscope for phonocardiography and ECG monitoring, using polymer-based dry electrodes (Fig. 19, Fig. 22) [26].

It is important to note that the majority of the prototypes [23], [24], [25], [26] mentioned beforehand use the purpose-built ScientISST firmware and Python API for biosignal acquisition, highlighting its efficiency in this type of tasks. The only exception was the smart chair [22], as this system includes a multiplexer to interface with the load cells’ ADCs, which requires an external code library to be controlled. Since the ScientISST CORE is compatible with the Arduino IDE, unlike some devices found in the state-of-the-art (Table 3), a custom firmware was developed in this IDE for this system.

Additionally, the plug-and-play nature of the ScientISST CORE device makes it extremely easy to interface this board with external sensors for biosignal acquisition, as seen in the aforementioned prototypes, where both ScientISST [23], [26] and third-party [22], [24], [25], [26] sensors were used. For additional information on these prototypes, we refer the reader to [22], [23], [24], [25], [26].

### 7.7 Conclusion

In this work we have introduced and characterized experimentally ScientISST CORE, a low-cost highly optimized hardware platform, designed to empower students, researchers or anyone with an interest in developing projects involving biosignal acquisition with a hardware tool to easily create microcontroller-based devices, and bring their health and well-being creations to life. By default, the board comes pre-programmed with a firmware optimized for real-time data acquisition and streaming, and can be used seamlessly with the available open-source software and APIs. In addition, it is also compatible with the Arduino IDE, enabling users to develop their own custom firmware.

Existing low-cost hardware platforms found in the state-of-the-art targeting physiological computing have had limited evolution when considering the latest developments in embedded systems. Commonly used hardware platforms are not particularly well designed to deal with the stringent requirements of biosignal acquisition (e.g. Arduino), whereas others that do meet these requirements have been discontinued, are too expensive, or have some limitations that make them unsuitable for certain biosignal-related applications. Our work establishes a new gold standard tool for biosignal sensing, overcoming the existing limitations for biosignal acquisition (e.g. increased ADC bit-resolution, higher sampling rates, etc.).

With the goal of attesting the performance of this system in terms of real-time data acquisition and streaming, tests were conducted to evaluate the throughput, the temporal accuracy of the ADC process, and the effect of battery discharge during operation. Our results show that ScientISST CORE was capable of reliably and accurately performing real-time signal acquisition with no loss of data at sampling rates of up to: 1 kHz for all analog channels; 2 kHz for all external analog channels; 8 kHz for a single external analog channel; and 10 kHz for a single internal analog channel. Additionally, using sampling rate values which cannot divide the base clock frequency of the ESP32 MCU module by a whole number of cycles was found to lead to large amounts of drift in the acquired signals. Thus, the use of the sampling rate values shown in Table 7, Table 8 and their factors are recommended. Lastly, the battery discharge analysis showed that the voltage drop in the power supply did not affect signal acquisition until the battery voltage level dropped approximately below the board’s operating voltage before shut-off, guaranteeing accurate readings for the remaining time of operation.

Future work will be mainly focused on: Adding the option in the software APIs to record the real timestamp for each data packet, instead of deducing it from the selected sampling rate, and potentially include the drift compensation in these records already; Implementing new APIs in other programming languages (e.g. Javascript, C++); Including a 9-axis Inertial Measurement Unit (IMU) in the next version of the CORE board, to measure Euler angles, angular velocity and linear acceleration, which can be useful in a number of projects, particularly those involving human activity recognition, tracking or navigation tasks.

## CRediT authorship contribution statement

**Leonor Pereira:** Writing – review & editing, Writing – original draft. **Francisco de Melo:** Software, Methodology, Conceptualization. **Frederico Almeida Santos:** Validation, Software, Formal analysis. **Afonso Fortes Ferreira:** Validation, Investigation. **Hugo Plácido da Silva:** Writing – review & editing, Validation, Supervision, Software, Resources, Project administration, Funding acquisition, Formal analysis, Conceptualization.

## Declaration of competing interest

The authors declare that they have no known competing financial interests or personal relationships that could have appeared to influence the work reported in this paper.

## Footnotes

13 If SD card initialization fails, the data is transmitted to the API, using BT as a fallback.

14 https://scientisst.github.io/scientisst-sense-api-python/.

## Footnotes

1 https://docs.arduino.cc/hardware/uno-rev3.

2 https://docs.arduino.cc/hardware/mkr-wifi-1010.

3 https://pulsesensor.com/.

4 http://www.advancertechnologies.com/p/myoware.html.

5 http://www.my-signals.com/.

6 https://scientisst.com.

7 https://www.espressif.com/sites/default/files/documentation/esp32-wroom-32_datasheet_en.pdf.

8 https://www.espressif.com/sites/default/files/documentation/esp32_datasheet_en.pdf.

9 https://wiki.seeedstudio.com/Grove_System/#-grove-cables-.

10 https://docs.espressif.com/projects/esp-idf/en/latest/esp32/#.

11 https://github.com/scientisst/scientisst-sense-api-python.

12 https://www.kicad.org/.

15 https://docs.espressif.com/projects/esp-idf/en/latest/esp32/get-started/##step-1-install-prerequisites.

16 https://www.tek.com/en/datasheet/afg3000c-arbitrary-function-generator-datasheet.

17 https://www.tek.com/en/datasheet/mixed-domain-oscilloscopes.

18 https://www.eembc.org/coremark/index.php.
